# Collaborative augmented reconstruction of 3D neuron morphology in mouse and human brains

**DOI:** 10.1038/s41592-024-02401-8

**Published:** 2024-09-04

**Authors:** Lingli Zhang, Lei Huang, Zexin Yuan, Yuning Hang, Ying Zeng, Kaixiang Li, Lijun Wang, Haoyu Zeng, Xin Chen, Hairuo Zhang, Jiaqi Xi, Danni Chen, Ziqin Gao, Longxin Le, Jie Chen, Wen Ye, Lijuan Liu, Yimin Wang, Hanchuan Peng

**Affiliations:** 1 New Cornerstone Science Laboratory, SEU-ALLEN Joint Center, Institute for Brain and Intelligence, Southeast University, Nanjing, China; 2 Guangdong Institute of Intelligence Science and Technology, Hengqin, China; 3 School of Computer Engineering and Science, Shanghai University, Shanghai, China; 4 School of Future Technology, Shanghai University, Shanghai, China

**Keywords:** Computational biology and bioinformatics, Neuroscience

## Abstract

Digital reconstruction of the intricate 3D morphology of individual neurons from microscopic images is a crucial challenge in both individual laboratories and large-scale projects focusing on cell types and brain anatomy. This task often fails in both conventional manual reconstruction and state-of-the-art artificial intelligence (AI)-based automatic reconstruction algorithms. It is also challenging to organize multiple neuroanatomists to generate and cross-validate biologically relevant and mutually agreed upon reconstructions in large-scale data production. Based on collaborative group intelligence augmented by AI, we developed a collaborative augmented reconstruction (CAR) platform for neuron reconstruction at scale. This platform allows for immersive interaction and efficient collaborative editing of neuron anatomy using a variety of devices, such as desktop workstations, virtual reality headsets and mobile phones, enabling users to contribute anytime and anywhere and to take advantage of several AI-based automation tools. We tested CAR’s applicability for challenging mouse and human neurons toward scaled and faithful data production.

## Abstract (web summary)

Collaborative augmented reconstruction (CAR) is a platform for large-scale reconstruction of neurons and other cells from multi-dimensional imaging datasets. It can be accessed from a variety of devices simultaneously for efficient and accurate reconstruction.

## Main

Three-dimensional (3D) neuron morphometry offers direct insights into the complex structures and functions of individual neurons and their networks, enhancing our understanding of the brain and its capabilities^1–4^. Morphometric measurements of neurons, particularly at the single-cell level and throughout an entire brain, have garnered several seminal datasets including several thousand fully reconstructed neurons in mouse brains^5–7^. The generation of these morphology datasets became possible thanks to both advances in sparse neuron labeling and light microscopy imaging of whole brains^8–16^ and particularly the development of neuron-reconstruction (also called neuron-tracing) technologies for 3D light microscopy images^17,18^.

A goal of neuron-reconstruction methods is to reconstruct digital models of the complete neuronal morphology with a low error rate^17–22^. Neuron-tracing techniques can be categorized as manual, semi-automatic and automatic methods, and varying levels of automation enabled by computer algorithms and the manual involvement of human labor impact the efficiency and the productivity of the digital reconstruction. The current convention for obtaining accurate neuronal reconstructions on a large scale primarily relies on manual labor-dominant methods^5–7^. While some attempts have integrated multiple repeated annotations for the purposes of correcting potential subjective errors from individual annotators and achieving higher precision, the overall efficiency could still be improved^23–25^. Despite a number of successes in automated neuron tracing, the majority of automation has only been applied to fairly simple use cases in which the signal-to-noise ratio is high or the entirety of neurite signal is not required to be traced^17^. Indeed, as the community has recognized that there is no single best algorithm for all possible light microscopy neuronal images^26,27^, careful evaluation of automated tracings must be cross-validated before they may acclaim biological relevance^18^. Therefore, a key question in the field is how to produce 3D reconstructions of complicated neuron morphology at scale while ensuring that these reconstructions are both neuroanatomically accurate and reliable.

We believe that the ultimate achievement of large-scale neuron morphology production will entail harnessing automation algorithms and increasingly powerful computing hardware to augment data-production rates within specified time frames. To reach such a goal, we considered practical challenges that must be surmounted. Neuron morphology encompasses a multitude of delicate characteristics, including the presence of thin yet extensive neurite fibers and spines as well as intricate broken signal patterns along neurites caused by the uneven distribution of light indicators (for example, fluorescent proteins) during the neuron-labeling process^20^. It is imperative to exercise caution to prevent unintentional compromise of these structures throughout tracing and preliminary processing steps, such as image preprocessing^28–31^. In addition, neurons frequently possess complex structures that can hinder the attainment of unequivocal observations. This complexity can become magnified when a region contains multiple neurons, and large projecting neurons need to be reconstructed from whole-brain images that contain trillions of voxels. Due to these hurdles, high-quality training datasets of neuron morphology are currently scarce, making the development of deep learning and similar machine learning methods for this task a formidable challenge^17^. A practical approach to leveraging learning-based techniques for neuron reconstruction involves identifying critical topological structures of neurons, such as branching points and terminal points^32,33^. However, without human validation, the results generated by these methods may still lack biological relevance.

While the challenges in neuron reconstruction are substantial and cannot yet be fully addressed through pure AI approaches, we have taken a proactive step toward overcoming these hurdles. We developed the CAR platform to enable many annotators and end users to contribute to annotating complicated 3D morphology in a collaborative way, which at the same time is also enhanced to generate neuroanatomically plausible reconstructions by leveraging specifically designed AI tools to automate data production to achieve precision and completeness in neuron morphology. We have integrated collective intelligence with AI for the task of neuron reconstruction from large-scale 3D brain images, resulting in a human-in-the-loop design that boosts both the biological relevance of the reconstructed morphology and the production speed.

Here, we showcase CAR’s effectiveness in several applications for challenging mouse and human neurons toward scaled and accurate data production. Our data indicate that the CAR platform is suitable for generating tens of thousands of neuronal reconstructions used in our companion studies^34^. We have adopted CAR as a major morphological data-generation platform in several ongoing projects including the BRAIN Initiative Cell Census Network and BigNeuron^18^.

## Results

### The CAR platform enables versatile morphometry in real time

**Fig. 1:**
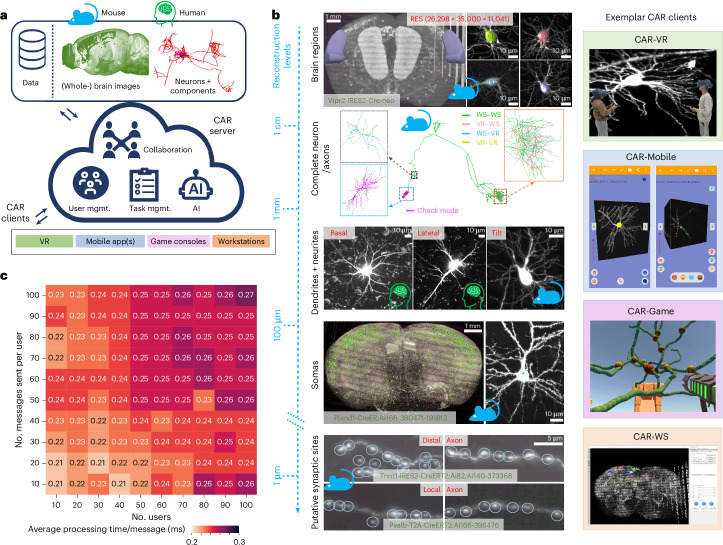
The CAR platform and its neuroscientific applications. **a**, The framework of the CAR platform. CAR has a cloud-based architecture and supports diverse types of clients, including workstations, virtual reality (VR) tools, game consoles and mobile apps. CAR is built upon a comprehensive collaboration mechanism, provides management (mgmt.) for data, tasks and users and is boosted by AI capabilities. The system is focused on mouse and human brain data. **b**, Left, typical morphometric tasks performed using CAR at different reconstruction levels from centimeter to micrometer scales, for example, brain region location tagging, complete neuron reconstruction (WS–VR, neurites annotated by workstation (WS) and validated by VR; WS–WS, VR–WS, VR–VR, similar meanings; check mode, neurites that are reconstructed yet validated), neurite tracing, soma annotation, bouton detection and proofreading. Right, example CAR clients are showcased, including CAR-VR, CAR-Mobile, CAR-Game (also called BrainKiller, unpublished work) and CAR-WS. **c**, The performance of the CAR server under concurrent scenarios. In CAR, the annotation operations were synchronized among the server and the users using network messages. We modeled situations where many collaborating users (ranging from ten to 100) were sending a burst number of messages. The heatmap shows the average processing time at the CAR server for each message. The *y* axis indicates the number of messages sent per user, while the *x* axis represents the number of users engaged in concurrent tasks.

Our major result in this study is to develop a CAR platform for the challenges of 3D neuron reconstruction from noisy, large 3D light microscopy images of mammalian brains. Compared to other neuron-reconstruction software packages, CAR stands out as a versatile computational platform (Supplementary Tables 1 and 2). It was designed to address the challenges associated with faithful reconstruction of neuronal images in mammalian brains, with a particular emphasis on mouse and human brains. CAR’s scalability also allows it to cater to a wide range of neuroscience applications (Fig. 1). One key strength is its accessibility across various client-end devices with built-in AI components, including regular desktop and laptop computers, virtual reality (VR) headsets, mobile phones and game consoles. This device compatibility enables efficient visualization and annotation of intricate 3D neuroscience data (Fig. 1a,b and Supplementary Fig. 1). These diverse client options offer advantages for neurodata validation by providing evidence of its completeness and accuracy. With the CAR platform, we organized a geographically dispersed team to collaborate effectively, as shown in several applications below. Team members worked together in real time within a shared virtual environment, allowing them to view and interact with each other’s annotations promptly, with the assistance of real-time AI-powered tools. CAR also offers the flexibility for users to work independently while maintaining data synchronization among the team, fostering seamless collaboration.

**Extended Data Fig. 1:**
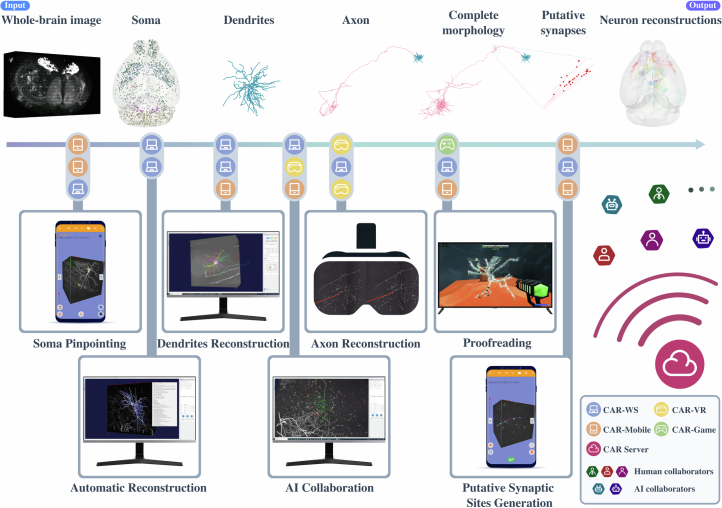
A comprehensive workflow of using various components of CAR for the task of reconstructing complete neuronal morphology from whole-brain images. Starting from whole-brain images, the CAR platform enables the gradual generation of morphological data, including soma, dendrites, axon, boutons, and more, culminating in the production of complete neuron reconstructions (upper row). During the process, a series of tasks, such as soma pinpointing, automatic reconstruction, dendrites tracing, axon tracing, AI-collaborated quality checking, topology proofreading, and putative synaptic sites generation, can be flexibly carried out in a collaborative manner (bottom rows). Users can select their preferred CAR components for each task (the icons along the time axis), while all the operations are coordinated by the CAR server (middle right). Bottom right: legends.

**Extended Data Fig. 2:**
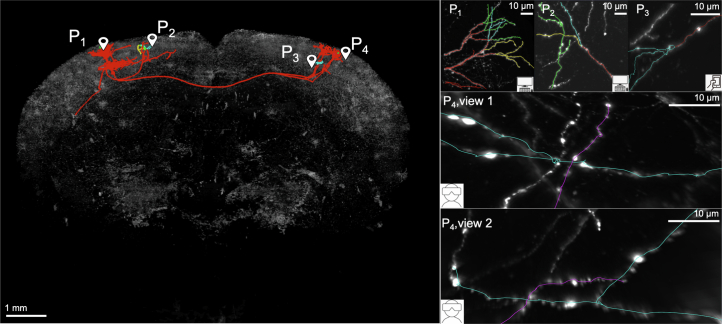
Example collaborative neuron reconstruction from whole-brain data using CAR. In this example collaborative effort, five users positioned at four locations (one user each at P1, P2, and P3, with two users at P4) utilized three types of CAR clients (desktop workstation, VR, and mobile app) to collectively reconstruct a neuron. The left panel provides a global view, while the right panel offers local perspectives. In all panels, neurites that have undergone proofreading are highlighted in red, while unchecked neurites are depicted in user-specific colors. Specifically, two users worked at P1 and P2, employing desktop workstations to reconstruct neurites. Meanwhile, the user at P3 inspected others’ reconstructions using the mobile app. At P4, two users wearing VR headsets collaborated to determine whether two adjacent neurites formed a bifurcation or not.

We used CAR to investigate brain anatomy across various scales. Specific tasks include tagging 3D brain regions, reconstructing entire neurons, tracing local dendritic and axon arbors, identifying somas, verifying potential synaptic sites and making various morphometric measures (Fig. 1b and Extended Data Fig. 1). These tasks often necessitated collaboration among team members who used different types of CAR clients. For instance, five users used CAR and collaborated to reconstruct a complete neuron from whole mouse brain imaging data, with different clients focusing on reconstruction of terminal branches and middle branches and bifurcation or a crossing (Extended Data Fig. 2). CAR offers the flexibility for a team of collaborators to engage in multiple reconstruction tasks for the same dataset concurrently, and it also integrates support from automation modules (Supplementary Fig. 2). Furthermore, game consoles were employed to validate the topological accuracy of the reconstruction. As CAR benefits a team with enhanced productivity and communication, CAR facilitates comprehension of complex neuron structures and knowledge sharing among users who might be geographically dispersed.

CAR’s cloud server manages centralizing operations, synchronizing annotation data and resolving any conflicts that may arise (Fig. 1a). All data, including 3D microscopic brain images and reconstructed neuron morphology, are hosted in cloud storage; therefore, users do not need to maintain data locally at CAR clients. We found that the CAR server was capable of handling large numbers of users and message streams in real time. Indeed, the CAR server responded within 0.27 ms even for 10,000 concurrent messages (Fig. 1c).

### Projection neuron reconstruction with converging correctness

We tested CAR on challenging 3D annotation tasks that encompassed large, multi-dimensional datasets. In the first application, we used CAR to annotate complicated 3D morphologies of large projection neurons in whole mouse brains, where a typical testing dataset involves an *xyz* volumetric brain image with about 40,000 × 30,000 × 10,000 voxels, or 12 teravoxels. CAR allows us to annotate an initial neuron morphology reconstruction that has been generated either using an automatic neuron-tracing algorithm or from scratch. Large-scale reconstruction is achieved through a series of CAR components, including CAR-WS and CAR-VR, which have robust large data-handling capabilities.

**Fig. 2:**
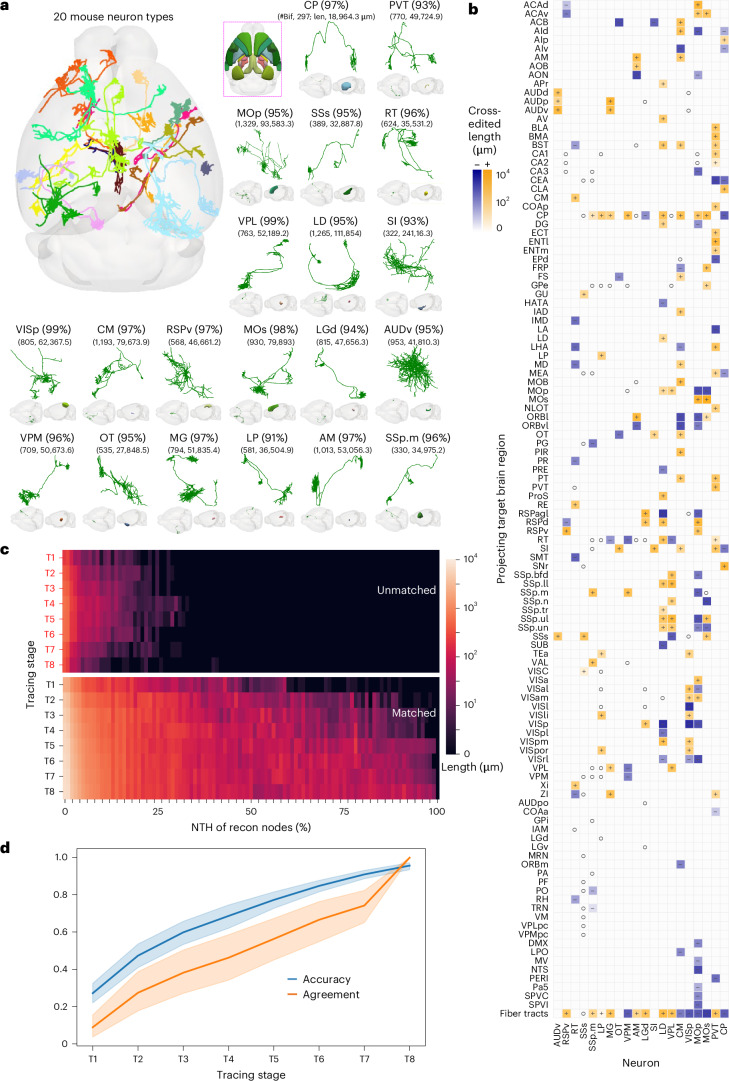
Reconstruction of complete mouse neurons using CAR. **a**, Complete reconstruction of example mouse neurons from 20 different brain regions. Top left, top–down view of example neurons registered to the standard Allen Brain Atlas. Each color represents an individual neuron, and the inset on the right indicates the respective brain region to which these neurons belong. Bottom and right, visualization of the neurons separately, providing their type, reconstruction accuracy, number of bifurcations (#Bif) and total length (len; μm). The mapped morphology in the standard atlas and the brain region that the neuron originates in are also visualized below each neuron. AM, anteromedial nucleus; AUDv, ventral auditory area; CM, central medial nucleus of the thalamus; CP, caudoputamen; LD, lateral dorsal nucleus; LGd, lateral geniculate complex, dorsal part; LP, lateral posterior nucleus; MG, medial geniculate complex; MOp, primary motor area; MOs, secondary motor area; PVT, paraventricular nucleus; RSPv, ventral part; RT, reticular nucleus; SI, substantia innominata; SSp.m, primary somatosensory area, mouth; SSs, supplemental somatosensory area; VISp, primary visual area; VPM, ventral posteromedial nucleus. **b**, Projection map illustrates the lengths of reconstructed neurites contributed through collaborations. The horizontal and vertical axes represent the origin (soma location) and destination (projection location) regions, respectively. Each cell in the map represents a projection pair, with the darkness of shading corresponding to the amount of the cross-edited length by collaboration. A ‘+’ symbol (yellow) is employed to denote cases in which collaborative addition was the predominant operation, while a ‘−’ symbol (purple) is used for instances in which collaborative subtraction dominated the editing process. The symbol ‘o’ indicates that no editing was performed through collaboration. The detailed names of the brain regions can be found in Methods. **c**, NTH of recon nodes of matched and unmatched structures for 20 neurons. This plot compares the topological height of reconstructed nodes with expert results at eight stages along the reconstruction timeline. Matched structures (bottom) indicate successful reconstructions that align with expert results, while unmatched structures (top) deviate from expert results. **d**, Average accuracy and user consistency for 20 neurons across eight tracing stages. Blue polyline, average accuracy (blue shading, 95% confidence interval); orange polyline, agreement among the contributors (orange shading, 95% confidence interval). Source data

We focused on representative neuron types in the mouse brain, with the cell bodies situated in 20 anatomical regions corresponding to major functional areas, including the cortex, the thalamus and the striatum (Fig. 2a). These neurons form a broad coverage in the brain with often long axons (Fig. 2a). They also have variable 3D morphology in terms of projection target areas, projection length (about 1.90 cm to 11.19 cm) and complexity in their arbors (with about 300 to 1,300 bifurcations) (Fig. 2a). With the aid of CAR, we achieved reconstruction accuracy of over 90% for all test neurons (Fig. 2a), accomplished with the collaborative efforts of citizen scientists and validated by additional expert gatekeepers.

As shown in an example of annotation of a ventral posterolateral nucleus (VPL) neuron carried out by six citizen scientists, in which all neurite segments were cross-validated (Supplementary Figs. 3–5), an additional expert neuroanatomist further examined the reconstruction and adjusted less than 2% of the neuron’s substructures in this case (Supplementary Fig. 4c), which indicates that the citizen scientists’ consensus was comparable with the expert’s annotation. When we visualized the heatmap of user participation in editing different regions of the neuron, it showed that the most intensive user collaboration happened around dendrites and distal axonal clusters that correspond to complicated branching structures (Supplementary Fig. 4d).

**Extended Data Fig. 3:**
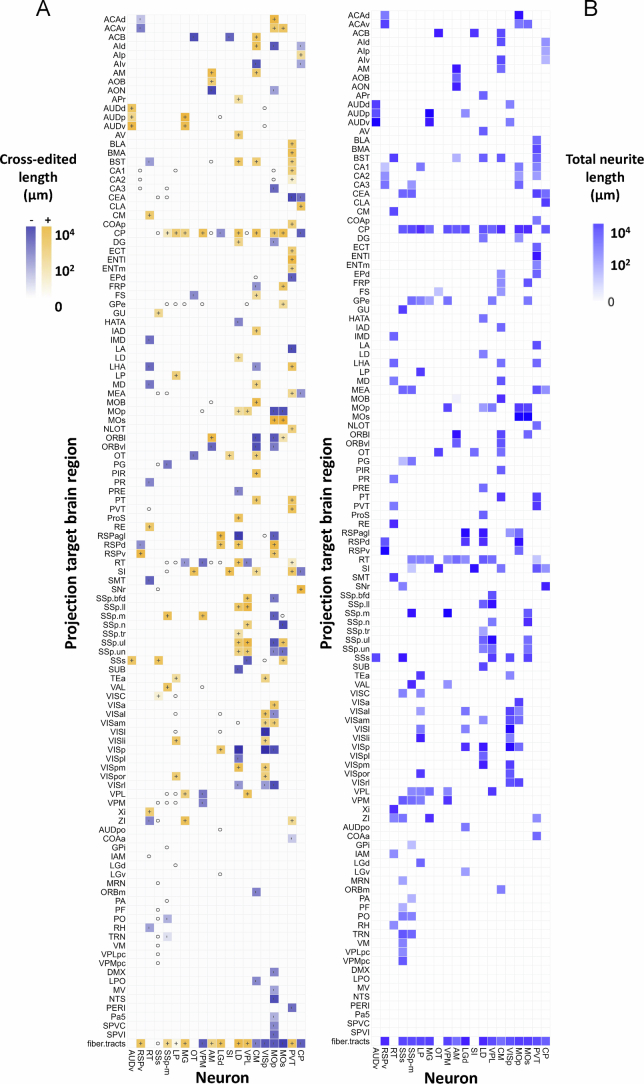
The projection maps formed by the 20 mouse neurons. **A**, A projection map derived from the collaboratively reconstructed sections of the 20 mouse neurons (identical to Fig. 2b, presented here again for comparison purpose). **B**, A complete projection map that encompasses reconstructions from both the collaborative and non-collaborative efforts. Color coding reflects the total neurite length within each specific region. Source data

Because the projecting targets of neurons hold essential information about their roles within the brain, we compared the projection maps derived from collaborative reconstructions and noncollaborative reconstructions performed by the same group of annotators. Through collaboration, we achieved a total neurite length of 84.8 cm for the 20 neurons. We also created a contrast map illustrating the edited differences between these two versions (Fig. 2b), revealing a total variation (including both additions and subtractions) in neurite length amounting to 37.3 cm. In other words, nearly 44% of the structures of these projection neurons underwent cross-editing (Extended Data Fig. 3). Notably, the noncollaborative version exhibited numerous instances of erroneously connected or missing neurites on the whole-brain datasets, which could considerably undermine subsequent analyses. In this context, the ability to cross-validate the reconstructions of projection neurons, as facilitated by the collaborative annotation approach of CAR, becomes crucial.

**Extended Data Fig. 4:**
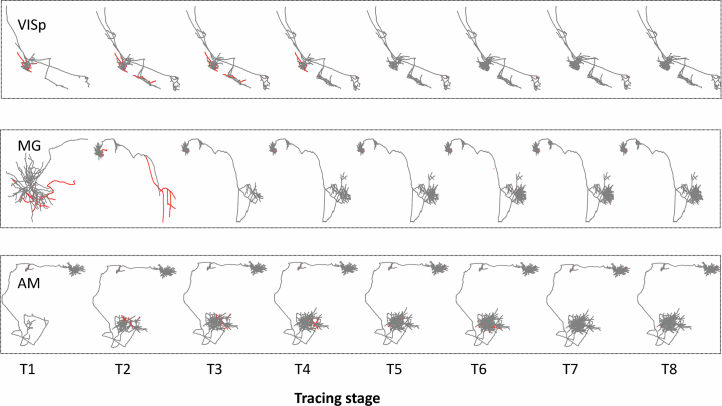
The morphologies across eight tracing stages for the reconstructions of VISp, MG, and AM neurons. Each row showcases a distinct neuron (VISp, MG, and AM), presenting its eight intermediate morphologies at time stages T1, T2,…, T8, arranged from left to right. Reconstructions in the early stages (for example, T1, T2) may be scaled up for enhanced clarity. Neurites shown in grey color represent correct structures that are matched with the expert-validated reconstructions, while neurites shown in red color represent unmatched structures.

An advantage of employing CAR is its capacity to identify potential unmatched (incorrect) reconstructions in a timely manner and avert unfavorable consequences. In other words, while errors may inevitably occur during the tracing of intricate neuronal arbors, this platform possesses the ability to limit potential errors and progressively refine the reconstruction process until a consensus is achieved among contributors. To facilitate quantitative analysis across different neurons, we defined a ‘normalized topological height’ (NTH) for reconstruction nodes within a neuron (Supplementary Fig. 6). NTH indicates the corrective effort required to rectify a reconstruction error involving a particular node and all its subsequent branching structures. The magnitude of the height directly correlates with the cost of modification. Across all tested mouse neurons, we observed a gradual reduction in the proportion of incorrect reconstruction components over both the tracing stage and the NTH (Fig. 2c and Extended Data Fig. 4). Notably, these errors remained confined to regions with low topological heights, suggesting that most reconstruction inaccuracies were rectified before they could give rise to further erroneous structures. In this way, CAR excels in both reconstruction accuracy and efficiency.

Finally, we observed a consistent enhancement in overall reconstruction accuracy toward greater than 90% as agreement among contributors steadily increased over time (Fig. 2d). CAR facilitates such collaboration, allowing each user to review other contributors’ reconstructions while simultaneously receiving assistance from fellow users.

### Branch and terminal classifiers for automated reconstruction

**Fig. 3:**
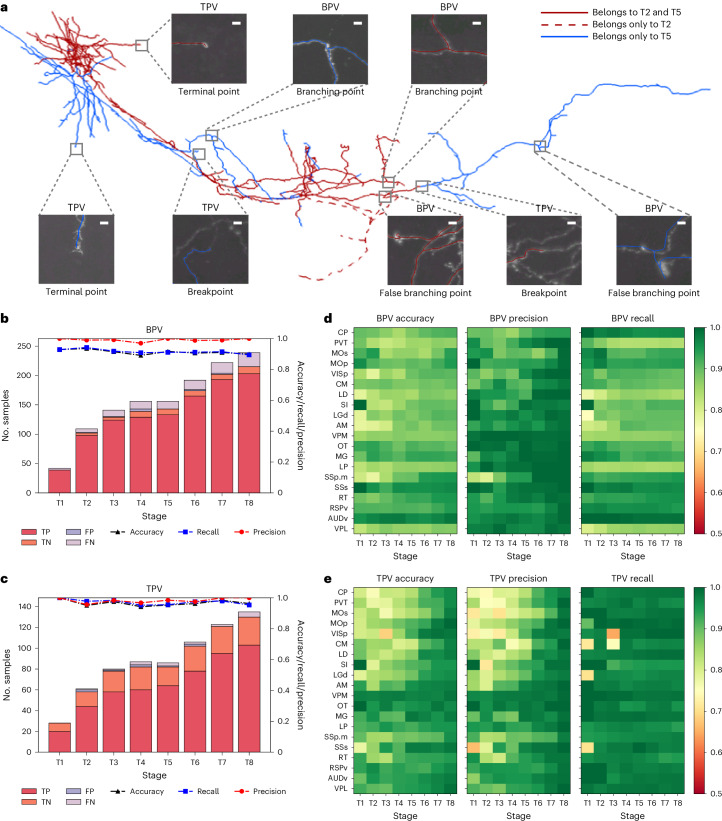
Collaboration of AI agents in CAR during reconstruction stages T1–T8. **a**, A halfway reconstructed OT neuron. Scale bar, 2 μm. The red-colored neurites (both in solid and dashed lines) comprise the morphology at T2, while the neurites shown in solid lines (both in red and blue) form the morphology at T5. Some incorrectly reconstructed parts in T2 are shown with dashed lines and were deleted by the time of T5, thanks to the hints provided by BPV. Subpanels show positive and negative cases of BPV and TPV, together with the image at the local region. **b**,**c**, The performance of BPV and TPV at eight stages, respectively. For each stage, the number of true positive (TP), true negative (TN), false positive (FP) and false negative (FN) samples is plotted as well as the accuracy, precision and recall. **d**,**e**, Accuracy, precision and recall of the two models for all 20 neurons at eight stages. Horizontal axis, stage; vertical axis, neuron type; color map, accuracy, precision and recall. Source data

One key feature of CAR is to augment the throughput of neuron reconstruction using two AI tools based on convolutional neural networks (Fig. 3 and Supplementary Fig. 7). First, a branching point verifier (BPV) was developed to determine whether the branching points in a reconstruction correspond to real bifurcation loci in the imaging data (Supplementary Fig. 7a). BPV combines the advantages of attention mechanism and residual blocks to extract distinctive neuronal image features. Second, a terminal point verifier (TPV) was designed to identify potential interruption in tracing neurites by classifying real neurite terminals against potential early termination in tracing (Supplementary Fig. 7b). To better distinguish terminal points and breakpoints that share similar features, TPV allows the network to learn more distinctive features. Both TPV and BPV were deployed at the CAR cloud server to periodically assess the neuron reconstructions, followed by pushing various suggestions of potentially erroneous terminal points and branching points to CAR clients. This AI-augmented interactive annotation was effective. Indeed, TPV and BPV behave like independent AI collaborators (contributors), frequently reminding human users to fix mistakenly reconstructed branching structures and continue tracing from forgotten breakpoints (Fig. 3a).

For instance, for the olfactory tubercle (OT) neuron (Fig. 2a), compared to the expert-validated reconstruction, the overall accuracy, precision and recall of both BPV and TPV were above 90% throughout the reconstruction process (Fig. 3b,c), even for partially completed neuron reconstructions. We also tested the applicability of both tools for other types of projection neurons that have many thin, often broken axonal branches (Fig. 2a). We observed that, during the entire reconstruction process, TPV and BPV consistently yielded an average accuracy over 90% and 85%, respectively (Fig. 3d,e). This means that our AI tools can reliably produce useful hints for human curation, largely independent of the completeness of reconstructions.

### Reconstructing human cortical dendrites in ten brain regions

In our effort to trace complete mouse neurons with long, faint projecting axons (Figs. 2 and 3) that are labeled using genetic and viral methods^11,35^, the dendrite-tracing parts of these neurons were not as difficult as the axon-tracing parts because of the relatively low noise level in the respective dendritic areas in mouse brain images. Here, we also applied CAR to reconstruct human cortical neurons where their dendritic images have abundant noise, due to various artifacts of dye injection, which is another widely used method for neuron labeling.

**Fig. 4:**
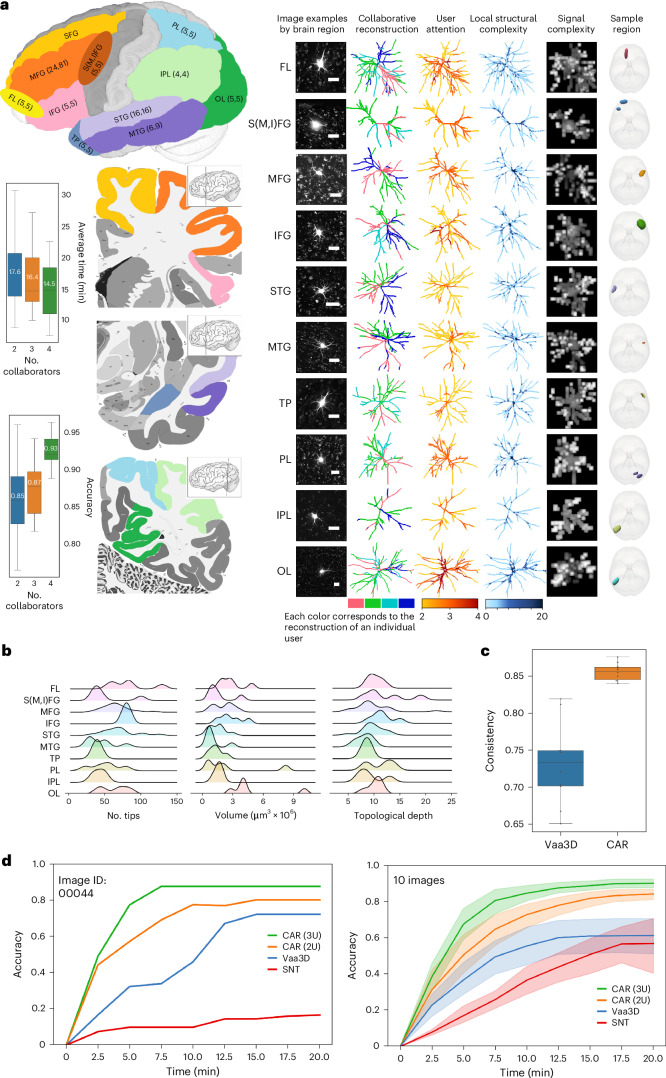
CAR used in human neuron reconstruction. **a**, Left, semantic mapping scheme of human brain regions reconstructed. Each brain region is represented by a different color. For each region, the two numbers indicate the number of individual neurons reconstructed and the total reconstruction copies (some neurons were reconstructed more than once for validation), respectively. Right, representative cells for different brain regions are displayed in each row. From left to right, each column gives the images (scale bar, 50 μm), user participation (each color corresponds to the annotation of an individual user), user attention heatmaps (color indicates the number of editing users for the region), local structural complexity (color indicates the complexity of the local structure), imaging signal complexity (intensity indicates signal complexity in the local region) and the actual locations where brain tissues were extracted, each represented by a different color. These extracted regions were then mapped and overlaid onto the standard brain coordinate template MNI152. Box plots on the left depict the relationship between reconstruction time and accuracy for various collaboration patterns across all neurons. The mean for each group is indicated by numbers in white. Medians are marked with a central black line. Each box represents the interquartile range, spanning from the 25th percentile to the 75th percentile of the data distribution for each group. Whiskers extend to the furthest points within 1.5 times the interquartile range from both the lower and upper quartiles. The data encompass results from three independent experiments with sample sizes *n* = 13 for two collaborators, *n* = 17 for three collaborators and *n* = 15 for four collaborators. FL, frontal lobe; IFG, inferior frontal gyrus; IPL, inferior parietal lobule; MFG, middle frontal gyrus; MTG, middle temporal gyrus; OL, occipital lobe; PL, parietal lobe; SFG, superior frontal gyrus; S(M,I)FG, an intermediate region bordering the SFG, the MFG and the IFG; STG, superior temporal gyrus; TP, temporal pole. **b**, Number of tips, volume and topological depth for the ten types of neurons. These three features are defined in L-Measure^52^. **c**, Consistency of reconstructions for both Vaa3D and CAR trials, involving a total of ten neurons (*n* = 10). Each neuron in the Vaa3D trials was annotated twice by two different users independently. Similarly, in the CAR trials, each neuron was annotated twice as well, with each annotation performed by a different group of two individuals working collaboratively. Box plot details are as in **a**, showing data quartiles, medians and outliers. **d**, Comparison of reconstruction accuracy over time among different tools: CAR with three users (3U), CAR with two users (2U), Vaa3D 3D viewer with one user and SNT with one user. On average, CAR (three users) achieved convergence within approximately 10 min. To enhance comparability, a 20-min upper time limit was applied to all tools tested, which is twice the convergence time of CAR (three users). Left, an example neuron (image ID 00044). Right, all ten neurons. Data are shown as mean ± 95% confidence interval. Source data

**Extended Data Fig. 5:**
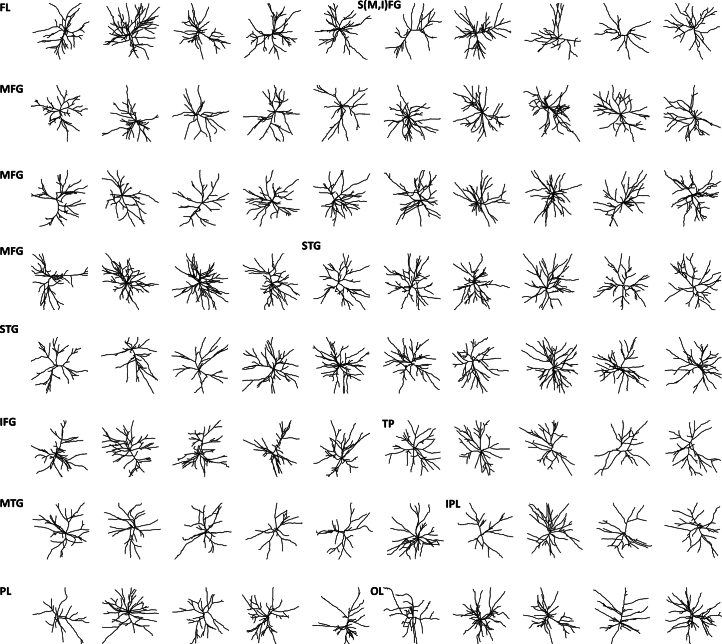
Display of 80 human neurons reconstructed using CAR. These neurons originate from 10 distinct brain regions, specifically S(M,I)FG, FL, MFG, IFG, STG, MTG, TP, PL, IPL, and OL. Region-names can be found in Methods.

We considered human cortical neurons generated by a consortium involving human neuron extraction, labeling, mapping, reconstruction and modeling using a human adaptive cell tomography method^36^. While human brain images can be obtained in high-throughput through perfusion and imaging, the noise level is substantial because of the fluorescence of blood vessels and dye leaking out of injected cell bodies or other injection sites. These human neuron images often fail in other neuron-tracing methods. We used CAR to reconstruct 80 human neurons from ten cortical regions (Fig. 4a and Extended Data Fig. 5). These neurons were mainly pyramidal cells with around 100 branches and ~15–20 topological layers of bifurcations embedded in images with intense noise (Fig. 4a,b). The reconstruction results showed that annotators effectively collaborated on reconstructing various parts of these neurons, especially focusing on areas with high branching density where the structural complexity was large (Fig. 4a).

As the number of collaborators using CAR increased from two to four, neurons were reconstructed with 7% to 18% less time, while the overall error decreased from above 15% to as little as 7% steadily (Fig. 4a). The collaboration of four contributors showed promise in reconstructing 15 randomly selected neurons with varying signal-to-noise ratios. Their combined effort yielded an accuracy rate of approximately 91% (Supplementary Fig. 8).

Thanks to its built-in immersive visualization capability and the collective consensus among annotators, CAR can generate more stable results than alternative approaches that do not optimize collaborative reconstructions. For instance, the intergroup consistencies in using CAR and Vaa3D’s 3D viewer^37^ were 0.86 and 0.73, respectively, when two groups of annotators were tasked with completing the same reconstruction (Fig. 4c). The advantage of CAR over conventional tools becomes even more pronounced when testing is performed on human neuron images with a high noise level. In the case of a randomly chosen testing image (no. 00044), CAR achieved an 85% accuracy of reconstruction within 7.5 min, whereas Vaa3D’s 3D viewer required 15 min but yielded inferior results (approximately 70% accuracy). Similarly, a two-dimensional (2D) visualization-based tool, SNT^38^, needed 20 min but still missed over 80% of neurites (Fig. 4d). In a comparison involving ten randomly selected neurons and expert-validated reconstructions, a consistent pattern emerged: CAR began converging to over 80% accuracy in about 7.5 min, whereas Vaa3D and SNT achieved a maximum accuracy of 50% to 60% at the expense of nearly double or triple the reconstruction time (Fig. 4d). Even when compared to the results obtained from the consensus of four reconstructions using conventional tools, CAR still demonstrates an advantage (Supplementary Fig. 9).

### Reconstructing somas and boutons at the whole-brain scale

In addition to generating reconstructions of complex axons and dendrites toward full neuron morphology as shown above, we also applied CAR to produce other types of digital reconstructions involving substructures of neurons at the whole-brain scale. One illustrative example is our application of CAR to detect somas in mouse brains. To do so, we first employed an automatic soma-detection algorithm to identify potential soma locations across diverse mouse brains and then used the CAR cloud server to dispatch image blocks containing the putative somas to many remote users who use CAR’s mobile client (Fig. 1). These users were able to fine-tune the soma locations in real time, cross-validated the results and completed annotation of each image block within a few seconds.

**Fig. 5:**
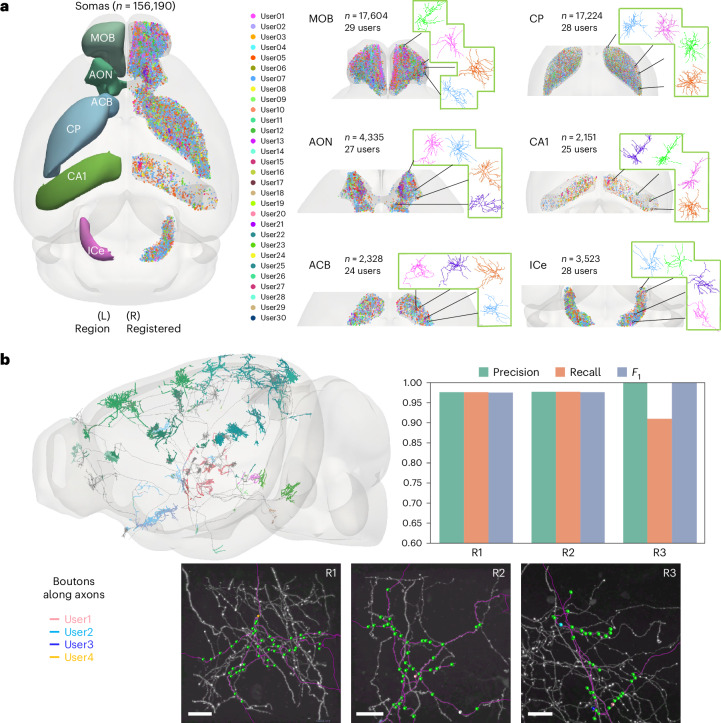
Reconstruction of subneuronal structures using CAR. **a**, Left, on the right side, a top–down view (CCFv3) of 156,190 semi-automatically annotated somas is presented, depicting six selected brain regions color coded along the anterior–posterior axis (left). Corresponding soma locations are plotted as dots (right). The different colors of the somas represent different users who contributed to the annotations. Right, close-up views of the eight selected regions mentioned in **a**, left. Auto-traced dendrites are also shown for those neurons for which somas have been annotated by different users. The six selected regions include the main olfactory bulb (MOB), anterior olfactory nucleus (AON), nucleus accumbens (ACB), the CP, field CA1 (CA1) and the inferior colliculus, external nucleus (ICe). The panel displays the number of annotated somas and users involved in the region. **b**, Top left, sagittal view of 20 neurons with boutons that were generated and proofread by CAR. Each distinct color represents boutons from different brain regions. Bottom, three image blocks (maximum intensity projection in 2D is shown), denoted as R1, R2 and R3, which were selected for evaluation (scale bar, 10 μm). Potential presynaptic sites that were auto-detected and validated are marked with green markers, while the white markers indicate incorrectly detected boutons that were deleted by users. Missing boutons spotted by the four users are shown in pink, azure, blue and yellow, respectively. Top right, the precision, recall and *F*_1_ scores for these three selected image regions. Source data

By employing this protocol, we generated one of the most extensive databases of annotated somas, using genetically labeled neurons across 58 whole mouse brains, which spanned a total of 609 brain regions, all aligned to the Allen Common Coordinate Framework (CCFv3; Fig. 5a). Specifically, we used the CAR-Mobile client to accurately identify 156,190 somas within approximately 4 weeks, involving collaboration among 30 users (23 trained users and seven novice annotators) (Fig. 5a). For the five most annotated brains, the annotation of each soma took only 5.5 s on average (Supplementary Fig. 10). Given the heightened precision of soma locations validated through the CAR-Mobile client compared to that of the initial automated detection, we were able to proceed with the further reconstruction of complicated neuronal morphologies within specific brain regions, such as the hippocampus and the striatum (Fig. 5a), still based on the CAR platform.

An advantage of using the CAR platform lies in its ability to streamline complex brain image analysis, spanning from the whole-brain scale down to the level of synapses connecting neurons. This advantage is exemplified in the case of whole-brain axon tracing (Fig. 2), followed by the detection of axonal boutons, which are the potential presynaptic sites of neuron connections (Fig. 5b). These boutons frequently manifest as concentrated varicosities arranged along axonal tracts, exhibiting an uneven distribution pattern^34,39^. Detecting or validating boutons directly, without any spatial constraints, would pose a formidable challenge^20^. However, CAR’s precise axon tracing and reconstruction of spherical image objects, such as somas, alleviate the challenge of bouton validation. The guidance provided by neurite fibers lends valuable cues for confirming boutons, and these structures can also be visualized using CAR’s toolkit. Consequently, we examined brain-wide bouton distribution in conjunction with fully reconstructed neurons (Fig. 5b). We randomly selected three image regions, each sized at 256 × 256 × 256 pixels (117.76 × 117.76 × 512 μm), designated as R1, R2 and R3, each containing both axon tracts and numerous boutons that were verified together by four individuals using CAR. Both the precision and *F*_1_ scores exceeded 0.9, affirming the suitability of CAR for comprehensive, large-scale analytics of whole-brain morphometry.

## Discussion

While recent endeavors have showcased achievements in acquiring thousands of complete mouse neuron morphologies^5,6^ and developing valuable software tools in the process^37,40–43^, the task of generating high-quality morphometry on a large scale remains a challenge yet to be fully resolved. In particular, establishing the accuracy of neuron morphology is a complex endeavor, owing to the inherent intricacies of neurons and the potential impact of individual annotator biases^44,45^. Many existing tools cannot produce accurate results due to their design involving observation of partial data in lower-dimensional space (for example, 3D data displayed as 2D series), partial observation of complicated data (for example, pseudo but not immersive 3D observation of neuronal structures that have complex branching structures), lack of multi-dimensional tools for cloud-based interactive annotations involving multiple annotators, etc. Within our study, we confront this challenge by introducing CAR, a tool designed to foster collaboration and facilitate the rectification of morphological and topological errors. Our tool achieves reconstructions that not only align with biological realities but also garner consensus among collaborators. Although there were efforts to develop collaborative tools^23–25,46–48^, most of them were designed specifically for annotating 2D image sections. In addition, simultaneous annotation was rarely adopted in prior collaborative tools. Through its provision of immersive interaction and collaborative editing of neuron anatomy, CAR empowers researchers to collaborate, capitalizing on their combined knowledge and expertise in solving challenges.

Queries regarding the efficacy of a multi-party collaboration within a multi-dimensional space to enhance tasks are deserving of further investigation. The MouseLight project^5^ adopted a fragment-connecting approach to assemble neurites into connected morphology, followed by generating the consensus results of independent human annotations using computer programs. FlyWire^47^ endeavored to collaboratively proofread neural circuits using a browser-based interface with spatially chunked supervoxel graphs. However, the performance of the browser-based interface could present potential challenges and limited scalability when handling extensive datasets. By contrast, the CAR framework incorporates a range of heterogeneous devices, including personal computers, VR headsets and mobile phones, each offering distinct advantages tailored to specific tasks, with the capability of intercollaboration supported by the CAR cloud server. Mobile clients are particularly suited for lightweight tasks, offering convenient data-visualization and -sharing capabilities and making them suitable for users needing mobility and quick validation of partial neuronal features. VR platforms, on the other hand, excel in tackling intricate neuron-annotation tasks, such as reconstructing neurons characterized by varying image quality and densely clustered structures in noisy images. The inclusion of a game console adds an interactive, gamified element that engages users and motivates increased involvement in the reconstruction process.

CAR integrates AI tools like BPV and TPV, as topological correctness and structural completeness are among the most crucial benchmarks for neuron reconstruction. This streamlined workflow substantially reduces the time and effort required for precise annotation without compromising the biological authenticity of the reconstructed morphologies. However, it is useful to validate results produced by AI models with human annotations. Instead of solely introducing individual AI models for profiling neuronal morphometry^32,33,49^, CAR offers a framework that enables collaboration among such AI agents and human contributors, ensuring that AI-generated results undergo thorough validation by a collaborating team. The framework of CAR further facilitates extension in the future by integrating more collaborating components such as AI-based skeletonization or fragment-connecting or consensus-generation algorithms.

Notably, Woolley et al.^50^ present empirical evidence highlighting the emergence of a collective intelligence factor in group collaboration. The study underscores the idea that a group’s collective intelligence is not solely tethered to the individual intelligence of its members. These findings carry substantial implications for comprehending group dynamics and efficacy. When we developed CAR, we noted that drawing a comparison between crowd wisdom and individual decision making could yield several key insights. While individual decision making can be susceptible to biases and a limited perspective, crowd wisdom amalgamates diverse viewpoints, mitigating individual biases and offering a more encompassing perspective conducive to accurate judgments and solutions. However, we also note that crowd wisdom does not guarantee superior outcomes across all scenarios. Factors such as groupthink, undue reliance on popular opinion, lack of diversity and suboptimal group dynamics can undermine its efficacy. Hence, cultivating an environment that nurtures diverse thinking, balanced participation and positive social dynamics becomes imperative for successful engagement with crowd wisdom.

Looking into the future, we envision broader applications for CAR while benefiting from an array of AI tools. These encompass intricate cell typing paradigms^6,14^ and the potential establishment of connectomes through the utilization of light microscopic brain images^51^.

## Methods

### Mouse brain region abbreviations

ACAv, anterior cingulate area, ventral part; ACB, nucleus accumbens; AId, agranular insular area, dorsal part; AIp, agranular insular area, posterior part; AIv, agranular insular area, ventral part; AM, anteromedial nucleus; AOB, accessory olfactory bulb; AON, anterior olfactory nucleus; APr, area prostriata; AUDd, dorsal auditory area; AUDp, primary auditory area; AUDv, ventral auditory area; AV, anteroventral nucleus of thalamus; BLA, basolateral amygdalar nucleus; BMA, basomedial amygdalar nucleus; BST, bed nuclei of the stria terminalis; CA1, field CA1; CA2, field CA2; CA3, field CA3; CEA, central amygdalar nucleus; CLA, claustrum; CM, central medial nucleus of the thalamus; COAp, cortical amygdalar area, posterior part; CP, caudoputamen; DG, dentate gyrus; ECT, ectorhinal area; ENTl, entorhinal area, lateral part; ENTm, entorhinal area, medial part, dorsal zone; EPd, endopiriform nucleus, dorsal part; FRP, frontal pole, cerebral cortex; FS, fundus of striatum; GPe, globus pallidus, external segment; GU, gustatory areas; HATA, hippocampo-amygdalar transition area; IAD, interanterodorsal nucleus of the thalamus; ICe, inferior colliculus, external nucleus; IMD, intermediodorsal nucleus of the thalamus; LA, lateral amygdalar nucleus; LD, lateral dorsal nucleus of thalamus; LHA, lateral hypothalamic area; LP, lateral posterior nucleus of the thalamus; MD, mediodorsal nucleus of thalamus; MEA, medial amygdalar nucleus; MOB, main olfactory bulb; MOp, primary motor area; MOs, secondary motor area; NLOT, nucleus of the lateral olfactory tract; ORBl, orbital area, lateral part; ORBvl, orbital area, ventrolateral part; OT, olfactory tubercle; PG, pontine gray; PIR, piriform area; PR, perireunensis nucleus; PRE, presubiculum; PT, parataenial nucleus; PVT, paraventricular nucleus of the thalamus; ProS, prosubiculum; RE, nucleus of reuniens; RSPagl, retrosplenial area, lateral agranular part; RSPd, retrosplenial area, dorsal part; RSPv, retrosplenial area, ventral part; RT, reticular nucleus of the thalamus; SI, substantia innominata; SMT, submedial nucleus of the thalamus; SNr, substantia nigra, reticular part; SSp-bfd, primary somatosensory area, barrel field; SSp-ll, primary somatosensory area, lower limb; SSp-m, primary somatosensory area, mouth; SSp-n, primary somatosensory area, nose; SSp-tr, primary somatosensory area, trunk; SSp-ul, primary somatosensory area, upper limb; SSp-un, primary somatosensory area, unassigned; SSs, supplemental somatosensory area; SUB, subiculum; TEa, temporal association areas; VAL, ventral anterior-lateral complex of the thalamus; VISC, visceral area; VISa, anterior area; VISal, anterolateral visual area; VISam, anteromedial visual area; VISl, lateral visual area; VISli, laterointermediate area; VISp, primary visual area; VISpl, posterolateral visual area; VISpm, posteromedial visual area; VISpor, postrhinal area; VISrl, rostrolateral visual area; VPL, ventral posterolateral nucleus of the thalamus; VPM, ventral posteromedial nucleus of the thalamus; Xi, xiphoid thalamic nucleus; ZI, zona incerta; AUDpo, posterior auditory area; COAa, cortical amygdalar area, anterior part; GPi, globus pallidus, internal segment; IAM, interanteromedial nucleus of the thalamus; LGd, dorsal part of the lateral geniculate complex; LGv, ventral part of the lateral geniculate complex; MRN, midbrain reticular nucleus; ORBm, orbital area, medial part; PA, posterior amygdalar nucleus; PF, parafascicular nucleus; PO, posterior complex of the thalamus; RH, rhomboid nucleus; TRN, tegmental reticular nucleus; VM, ventral medial nucleus of the thalamus; VPLpc, ventral posterolateral nucleus of the thalamus, parvicellular part; VPMpc, ventral posteromedial nucleus of the thalamus, parvicellular part; DMX, dorsal motor nucleus of the vagus nerve; LPO, lateral preoptic area; MV, medial vestibular nucleus; NTS, nucleus of the solitary tract; PERI, perirhinal area; Pa5, paratrigeminal nucleus; SPVC, spinal nucleus of the trigeminal, caudal part; SPVI, spinal nucleus of the trigeminal, interpolar part.

### Input and output for neuron reconstruction in CAR

The input for neuron reconstruction in CAR includes images of both mouse and human brains. The major distinctions between mouse and human brain images are outlined in Supplementary Table 3: CAR is mostly used for multi-dimensional LM images and is not limited to a specific image type. In practical applications, the image data are often first converted into a multi-resolution representation using tools such as TeraConverter^53^, especially if they are of a large scale (for example, containing trillion of voxels).

To work with one’s own data, a copy of the data can be stored locally on each user’s system as well as on the CAR server. Alternatively, a shared copy can be hosted on web data storage accessible by both the CAR clients and the CAR server. Virtually, there is no size limit for the image data, as long as there is sufficient storage.

The output of neuron reconstruction in CAR is a tree-like structure depicting the skeleton of the neuron, represented as nodes and edges and in either SWC^54,55^ or ESWC^56^ format. We employ a quasi-binary tree to represent neuronal morphology, with the exception that the soma node can have multiple children. With the morphological and imaging data, the radius of the traced neuron along the skeleton can be estimated in CAR-WS.

### A brief end-to-end neuron-reconstruction workflow using CAR

CAR offers a flexible collaboration framework, based on which a team of users can choose to use a range of clients to reconstruct neurons collaboratively. While there is not a fixed procedure or protocol for the task of neuron reconstruction using CAR, an illustrative workflow is given in Extended Data Fig. 1.

It commences with soma identification through CAR-Mobile. Initially, potential soma positions are automatically detected on the CAR server. Subsequently, users use the mobile interface to precisely label the position of the soma. For semi-automated and manual neuron-reconstruction tasks, users navigate through a 3D volume image, outlining the skeletal structure of the neuron in a 3D environment. Users have the flexibility to choose specific regions of interest with the desired level of detail on different device clients. Typically, a collaborative team works together, validating and refining each other’s reconstructions. Users can opt for auto-reconstruction algorithms (APP2) to enhance the efficiency of neuron reconstruction. When reconstructing axon neuron signals, especially for neurons with varying image quality and densely clustered structures, CAR-VR, which simulates stereo vision for immersive 3D visualization of neuron structures, can be employed to facilitate a clear understanding of the structures, particularly in challenging regions. During the neuron-reconstruction process, the AI modules on the CAR server periodically assess the reconstruction, inspecting annotations and placing marker points at potential error locations every 3 min. The users can then inspect these locations to decide whether there is an incorrect tracing.

Once the reconstruction is complete, it can be further sent to CAR-Game, where more users can validate the topological correctness of the neuron in a gameplay setting. For any suggested errors, user can continue to use CAR-WS or CAR-VR for necessary modifications. After the neuronal skeleton is finalized, a set of putative synaptic sites can be automatically generated. Users can use CAR-Mobile to perform further validation.

### Collaborative neuron-reconstruction protocol

To facilitate flexible and organized collaboration among CAR users, we have devised a straightforward neuron-reconstruction protocol (Supplementary Fig. 3). The protocol is underpinned by a set of rules governing the reconstruction process:

1. A user is permitted to annotate a neurite if it originates from one of the following: (1) the soma, (2) another neurite previously reconstructed by the same user or (3) a neurite that has been validated and confirmed.
2. Alternatively, users have the option to confirm, delete or modify neurites previously reconstructed by other users, provided that these neurites either originate from the soma or extend from another already confirmed neurite.
3. It is essential to note that a user is precluded from confirming their own reconstructions, emphasizing the importance of impartial validation.
4. The neuron-reconstruction process is considered complete only when all reconstructed neurites have been duly confirmed and there are no further unaccounted structures that can be added.

This protocol was designed for simultaneous annotation and cross-validation. Each user engages in annotating neuronal structures while also reviewing the reconstructions performed by other users during this process. Importantly, to resume tracing the neuron from a point where a fellow collaborator left off, the user must ensure that all the parent segments along the route are validated. In the presence of unexamined segments, the user should first verify their correctness and make any necessary adjustments before proceeding with further annotation. As a result, upon completion of a reconstruction, every segment in the neuronal tree has undergone cross-validation.

In the event of disagreement with the reconstruction of a neurite by user A, user B is permitted to make desired modifications. However, this modified annotation still requires confirmation from an additional user C. In cases in which obtaining a consensus is challenging, multiple users can inspect the region simultaneously, particularly using CAR-VR for unambiguous observation. By adhering to this protocol, we establish a robust framework for collaborative neuron reconstruction and verification. Annotations made by one annotator can be rigorously reviewed and endorsed by another annotator, thus bolstering the accuracy and the reliability of the overall annotation results.

### Artificial intelligence tools in CAR

Two AI-based tools are introduced in the user annotation process to assist users to achieve complete neuron reconstruction by identifying feature points including the branching points and the terminal points of neurons.

#### Implementation of BPV and TPV

To verify branching points, we designed a convolutional neural network called the residual single-head network (RSHN). The network consists of an encoding module, an attention module and two residual blocks. To reduce the dimensionality of the input, the patch undergoes an encoding process. The encoding operation is achieved by applying two 5 × 5 × 5 convolution kernels with a stride of 1, followed by two 3 × 3 × 3 convolution kernels with a stride of 2. After that, the network applies an attention module and residual blocks to extract salient features from the image patch. The residual block consists of two convolutional layers and one batch normalization layer. ReLU is used as the activation function for nonlinear processing. Finally, the output is obtained through a fully connected layer for classification (Supplementary Fig. 7a).

To differentiate terminal points and breakpoints, residual double-head networks (RDHNs), a variant of RSHN, are designed to process two inputs: an image patch and a corresponding mask image. The two images are separately encoded, and the resulting features are fed into the attention module for feature enhancement. The purpose of this is to emphasize the disparities between breakpoints and terminal points by obscuring the shared areas. This approach guarantees that the network acquires more distinguishing features and enhances its ability to differentiate between the two types of points (Supplementary Fig. 7b).

#### Training details

The two networks mentioned above are both developed using PyTorch, optimized using the stochastic gradient descent algorithm, supplemented by employment of the AdamW optimizer. The momentum parameters for the optimizer are set to the commonly used values of 0.9 and 0.999. The initial learning rate, set at 3 × 10^−4^, is first reduced to a tenth of its value after the third epoch and then decreases to a hundredth after the sixth epoch. Cross-entropy functions as the loss function throughout. The whole training is completed within 50 epochs.

During the neuronal reconstruction process with our software, we collect distinct datasets to develop two specialized networks: RSHN and RDHN. RSHNs, designed for verifying branching points, are trained on image patches centered on these points. Conversely, RDHN is focused on terminal point verification and trained using pairs of image patches and mask images centered on terminal points. The training dataset can be automatically acquired, with labels being a byproduct of the reconstruction process. During the user’s neuron reconstruction using CAR, new label data are automatically generated. The training dataset is generated by extracting critical points from existing completely reconstructed neurons. Intermediate results can also serve as valuable training data, often of higher quality, such as false branching point labels generated by the user at intermediate stages and deleted in the final result. As the user reconstructs more neurons, more high-quality labels are generated. Generally, a well-performing model can be trained with 10,000–20,000 critical points, which is equivalent to the data from ten to 20 whole brain neurons. To enhance the diversity and robustness of input images, we incorporate data-augmentation techniques such as rotation, cropping and flipping. Throughout this process, we maintain a balanced positive-to-negative sample ratio of 1:1.

#### The deployment of AI modules in CAR

In the process of deploying our AI models, we leverage Cog (https://github.com/replicate/cog), an open-source tool designed to streamline the deployment of models into standardized and production-ready Docker containers. The deployment process involves a series of well-defined steps:

1. Configuration: compose a cog.yaml configuration file to articulate the model’s environment dependencies.
2. Model definition: clearly define the model’s input–output format and outline the specific inference process.
3. Docker image building: build the Docker image using the provided configuration and model definition.
4. Container launch: initiate a Docker container based on the constructed Docker image. The container’s HTTP server is instrumental in providing an API for conducting model predictions.

#### The collaboration process of the AI tools

The CAR server maintains a comprehensive and progressive reconstruction morphology, periodically analyzing the latest annotated sections every 3 min. Critical points, including terminal and branching points, are identified during this process. The associated coordinates, along with the SWC file, are encapsulated into JSON format and transmitted to the AI system via an HTTP request.

The AI system framework is composed of specialized APIs for acquiring and updating neuronal reconstruction results as well as preprocessing input data through format conversion. Additionally, a BaseModel class is incorporated for model initialization and invocation. This modular design supports the integration of future algorithms and models, and it addresses the processing and the transformation of model output data.

Below is a list of the steps for the AI tools to work through to generate suggestions for users:

1. Initiate an HTTP request to retrieve the image resolution.
2. Prune tiny terminal branches with less than six units.
3. Conduct coordinate transformation for terminal points, branching points and skeleton files based on the obtained image resolution.
4. Use the coordinates of terminal or branching points as the center to send an HTTP request and acquire a 32 × 32 × 32-sized image, subsequently stored on the server.
5. Generate a mask image of the same dimensions based on the SWC file.
6. Combine the original image and the mask image, creating a unified two-channel 32 × 32 × 32-sized image serving as input for the model.
7. Perform inference using the model to obtain classification results, encapsulating them as JSON data for return to the AI system.
8. The AI system transmits the prediction results back to the collaborative server.
9. The collaborative server, guided by the results, sends corresponding coordinate point information to the client through socket connection.

#### Evaluation of BPV and TPV

To evaluate the accuracy of detection in the module, we designed the below metrics for assessment. In this context, the final expert-proofed reconstruction is designated as the ground truth. Candidate branching points are selected from the current reconstruction. These points serve as the central reference to extract image patches, which are subsequently used as inputs for the classifier. Similarly, candidate terminal points are chosen from all the terminations found in the current reconstruction. The image patches surrounding these points are also extracted and used as inputs to the classifier, with the corresponding breakpoints serving as centers. Based on measures (Supplementary Table 4), we have adopted the following three indicators for quantitative analysis, including precision, recall and accuracy. The three metrics are defined as follows:

$${\rm{Precision}}=\,\frac{{\rm{TP}}}{{\rm{TP}}+{\rm{FP}}},$$

$${\rm{Recall}}=\,\frac{{\rm{TP}}}{{\rm{TP}}+{\rm{FN}}},$$

$${\rm{Accuracy}}=\frac{{\rm{TP}}+{\rm{TN}}}{{\rm{TP}}+{\rm{TN}}+{\rm{FP}}+{\rm{FN}}}.$$

### Automatic neuron-reconstruction module

CAR can incorporate several components for automatic neuron tracing, which can be invoked either at the outset to generate an initial tracing or at any intermediate point to extend existing tracings. As an example, the APP2 algorithm^57^ has been integrated into CAR. Given a starting point, the APP2 algorithm can be invoked locally at a CAR client to automatically generate a local tracing. The tracing result is further appended to the existing reconstructions and synchronized among all the CAR users.

### Soma pinpointing in CAR-Mobile

The soma-identification protocol in CAR involves two major steps. The first step is the automatic detection of potential soma positions on the CAR server. The highest-resolution whole-brain images are partitioned into volumes with approximately 256 × 256 × 256 voxels. Subsequently, we filter out blocks with maximal intensities less than 250 (unsigned 16-bit image) and standardize the remaining blocks through *z*-score normalization, converting them to an unsigned eight-bit range. Following this, the blocks are binarized using their 99th percentile as thresholds, and the resulting images undergo transformation using the grayscale distance transform algorithm. Voxels with intensities in the range of 5 to 30 on the transformed image are identified as candidates and further processed using a non-maximal-suppression-based approach to eliminate redundant candidates. Image blocks (128 × 128 × 128 voxels) centered at potential soma positions are cropped and distributed from the CAR server to CAR-Mobile.

The second step involves pinpointing somas in the CAR-Mobile client. The process is carried out as follows:

1. The user clicks the ‘open file’ button in the mobile client.
2. The client sends a message to the server requesting potential location information.
3. The server checks the potential location table and selects an unprocessed location record for the client.
4. The client then sends a message to the server, requesting an image block centered around the location information with the appropriate size.
5. Additionally, the client sends another message to obtain existing soma positions within the bounding box of this specific block. It is important to note that image blocks may overlap when potential locations are close together; therefore, somas uploaded by other users may appear in the client’s block.
6. The server crops the image block size at 128 × 128 × 128 (typically encompassing one to five somas per block) from the whole-brain image based on the requested location and size.
7. The server also looks up the existing somas relevant to the client’s request. Clients are empowered to update soma information by making changes, additions or corrections to the identified soma data.

Each image is then randomly dispatched to two CAR users, with the first user proofreading the automation results and the second user verifying the result of the first user. CAR-Mobile facilitates efficient online collaboration. To prevent conflicts arising from simultaneous access to the same image, the CAR server implements a locking and expiration strategy. When an image is distributed to a client, the corresponding record in the table is locked, preventing the image from being distributed to other clients while the lock is active. The lock is automatically released when the client returns the annotation result or after a predefined period of 8 min. The results are sent back to the server and shared with other users for cross-validation. Each image block is disseminated to two individuals for this purpose. To enhance the browsing experience, CAR-Mobile uses a preload strategy. It maintains a queue of images, and a dedicated download thread ensures that the queue remains populated. When a user requests an image, the first image in the queue is retrieved, and any newly downloaded images are appended to the end of the queue. Each downloaded image has a predefined expiration time of 8 min from its initial download. Once expired, the client can no longer perform any actions with the image. This optimization strategy allows for efficient resource allocation and provides a smoother browsing experience within the CAR system.

### Putative synapse validation in CAR-Mobile

A putative synapse, or an axonal bouton, which is characterized as a localized swelling along axonal shafts, manifests as a region of high intensity in light microscopy data when observed at submicrometer resolution. The putative synapse-validation process bears similarities to the process of pinpointing somas in the CAR-Mobile client. It also consists of two steps:

First, we use an algorithm based on the approach presented by Liu et al.^34^ to automatically detect potential bouton positions. This method combines intensity and radius profiles along axonal shafts to identify initial candidates for boutons, characterized by overlapping peaks in both profiles. False positives are eliminated using heuristic criteria: boutons should be 1.5 times larger than surrounding nodes and have intensity values above 120 in eight-bit images and duplicate candidates closer than 5 voxels are discarded.

Next, we crop image blocks sized at 128 × 128 × 128 and their corresponding candidate boutons as well as morphology results. These blocks along with boutons and morphology results are distributed to clients, and users engage in proofreading tasks to identify and correct any missing or erroneous boutons within the image block. The validation results are sent back to the server and shared with other users for cross-validation. Each image block is distributed to two individuals for this purpose.

Second, the boutons are validated in the CAR-Mobile client. The boutons and the corresponding morphology results are integrated into CAR-Mobile clients for rendering. As a result, users only need to engage in proofreading tasks, identifying and correcting any missing or erroneous boutons within an image block distributed from the server. The validation results are then sent back to the server and distributed to other users for cross-validation. Each single image block is distributed to two individuals for this purpose.

### Post-reconstruction processing of morphological data

To analyze NTH values and the distribution and amount of axons in brain-wide targets, morphological data are examined and processed to ensure compatibility for downstream analysis. A single connected neuronal tree with the root node as the soma is obtained. Terminal branches with less than six units are pruned. Mouse neurons are then resampled and registered to CCFv3 using mBrainAligner^58^. Human neurons are also registered similarly.

### Analysis and evaluation of neuron reconstructions

#### Accuracy

Accuracy is computed as 2 × *R*_c_ × *R*_m_/(*R*_c_ + *R*_m_), where *R*_c_ is the ratio of the correctly traced length in the complete reconstruction and *R*_m_ indicates the ratio of the missing structures.

#### Agreement

Agreement denotes the ratio of the length of structures that have been mutually agreed upon. Agreed upon structures are those reconstructions that have been edited, examined and confirmed by at least two collaborators.

#### Recovering the reconstruction at a given time stage

We use the version control system of CAR to recover the neuronal reconstruction results at given moments. To analyze the structural patterns of the 20 neurons along the temporal dimension, we evenly divide each neuron’s reconstruction timeline into eight segments and recover reconstructions at the eight time stages. This approach allows us to analyze different neurons within the same temporal scale.

#### Normalized topological height

We consider topological height (TH) of the teminal nodes to be 1. As we traverse the neuron structure, the topological height of each branching point is determined by adding 1 to the highest level among its child nodes (Supplementary Fig. 6).

In the next step, we expand on this process by setting the maximum level observed among eight time stages as the denominator for calculating the topological height. The normalized topological height (NTH) is then defined as:

$${{\rm{NTH}}}_{{T}_{i}}=\frac{{{\rm{TH}}}_{{T}_{i}}}{\mathop{\max }\limits_{j\in [1,8]}{{\rm{TH}}}_{{T}_{j}}},$$

where a smaller level indicates a more peripheral position. This approach enables the comparison of forms across different time points.

Furthermore, under each NTH, we calculate the average length of both the matched and unmatched parts of 20 neurons at each time stage.

#### Computation of the contrast projection map

The reconstructions are performed only once through CAR collaboration. The noncollaborative results for comparison are calculated based on the collaborative ones, by assuming that the modifications made by one user to another’s annotations do not take place and by revoking the corresponding changes. The contrast projection map comprises two distinct components: the neurites that have been added through collaboration, denoted by the symbol ‘+’, and the neurites that have been subtracted through collaboration, represented by the symbol ‘−’. In detail, the addition projection map measures the contributions made by one user to complete the structures traced by other annoators. Meanwhile, the subtraction projection map represents the reconstructed neuron morphology that has been deleted by others. The full morphology projection analysis was performed using the Python package neuro_morpho_toolbox^6^.

#### Computation of morphological features

The morphological features of mouse brain neurons, including the number of bifurcations and the total length, were calculated using the Vaa3D plugin ‘global feature’.

#### User attention

The computation of user attention involves several steps. Initially, a cuboid region with dimensions of 20 × 20 × 20 μm^3^ is defined as a bounding box surrounding each neuron node. Within these regions, we collect the unique user editing that occurs. We focus on identifying distinct users and assign a color-shading-level attribute to each node based on the count of users. Darker colors signify higher attention levels, indicating increased user contributions to either the addition or the modification of the neuron segment.

#### Local structural complexity

Local structural complexity is a measure employed to quantify the intricacy of neuronal dendritic architecture within a specific region. It involves calculating the number of intersections at varying radial distances from each point in the structure, extending the traditional Sholl analysis concept by considering every point, not just the soma, as a center for assessing intersections with neighboring points.

#### Consistency

Consistency is quantified based on the distance between two distinct reconstructions of the same neuron. Specifically, distance is defined as the average distance between two neurons in all nearest point pairs. Given that the number of nodes can differ between pairs of reconstructions, distances are obtained twice using each reconstruction as a starting set for the search for nearest points in the other reconstruction. Finally, the average bidirectional distance is calculated. Together with the average distance, consistency is calculated as the percentage of nodes with pairwise distance less than two voxels for each of the compared reconstructions.

#### Consensus

The consensus of four reconstructions generated by Vaa3D and SNT for each image is calculated using the ‘consensus_skeleton_2’ algorithm from the BigNeuron project^18^. The consensus algorithm employs an iterative voting strategy to merge tracing results (SWC files) from different instances, selecting and connecting consensus nodes to create a unified representation.

### Image analysis

#### Signal complexity

To compute signal complexity, we use the reconstructed morphology of the neuron and estimated radius values as masks. Each voxel in the volume image is classified as either foreground or background based on these masks. Subsequently, the image is decomposed into a number of small cubes, for example, 20 × 20 × 20 voxels in size. The signal complexity of each cube is defined as the mean value of the foreground voxel intensity divided by the mean value of the background voxel intensity. Additionally, by uniformly converting the signal complexity values into the range of (0, 255), we can generate a specialized 3D image that visually represents the signal complexity of the original image.

#### Image quality assessment

To assess the image quality of the human neuron images in Supplementary Fig. 8a, an image decomposition method called non-negative matrix factorization (NMF) is used^59^. In the NMF decomposition process, the average of every ten image slices along the *z* axis is calculated and transformed into one-dimensional vectors. These vectors are collected into a matrix, on which a three-component NMF model is constructed to obtain the decomposed components representing the background and the signal obtained as the difference between the image block and the background component, enabling better separation between the two. After performing NMF decomposition, several metrics are calculated: (1) ‘signal.median’, this metric refers to the median value of the signal; (2) ‘signal.rsd’ (relative standard deviation), this metric is calculated by dividing the standard deviation of the signal by the median value of the signal; and (3) ‘contrast’, this metric is obtained by subtracting the median value of the signal from the median value of the background. During the calculation, only background values below the 99th percentile and signal values above the 90th percentile are considered. This helps to reduce the impact of residual signal in the estimated background and vice versa.

### Reporting summary

Further information on research design is available in the Nature Portfolio Reporting Summary linked to this article.

## Online content

Any methods, additional references, Nature Portfolio reporting summaries, source data, extended data, supplementary information, acknowledgements, peer review information; details of author contributions and competing interests; and statements of data and code availability are available at 10.1038/s41592-024-02401-8.

## Supplementary information

Supplementary Information Supplementary Note, Tables 1–4 and Figs. 1–10.

Reporting Summary

## Source data

Source Data Fig. 2 Statistical source data.

Source Data Fig. 3 Statistical source data.

Source Data Fig. 4 Statistical source data.

Source Data Fig. 5 Statistical source data.

Source Data Extended Data Fig. 3 Statistical source data.

## Data Availability

All data reported in this study, including mouse and human neuron reconstructions, soma locations and synaptic sites, are deposited at https://car.cvcd.xyz. Source data are provided with this paper.

## Code availability

CAR is open source and follows the Mozilla Public License version 2.0 protocol. Source code of CAR is publicly available on Zenodo (10.5281/zenodo.13103222)^60^ and GitHub (https://github.com/neurogeom/CAR). Additionally, binary executables and a user guide are available on the GitHub page.
