# Supplementary material for: Collaborative augmented reconstruction of 3D neuron morphology in mouse and human brains
Source: Nat Methods. 2024 Sep 4;21(10):1936–46. doi: 10.1038/s41592-024-02401-8 (PMC11468770; doi:10.1038/s41592-024-02401-8)
Supplement: Supplementary file 1 — Supplementary Note, Tables 1–4 and Figs. 1–10. [file 41592_2024_2401_MOESM1_ESM.pdf]

nature methods

Supplementary information https://doi.org/10.1038/s41592-024-02401-8

Collaborative augmented reconstruction of 3D neuron morphology in mouse and human brains

In the format provided by the authors and unedited

CONTENTS

1 CAR Clients..2
2 CAR Server..3
3 Programming environments..5
4 Hardware and environment configurations...5
5 Supplementary Tables..7
6 Supplementary Figures 11

1 CAR Clients

All the CAR clients are equipped to support the 3D visualization of images and annotations (as illustrated in **Supplementary Fig. 1**). Additionally, the display of 2D image sections is enabled in CAR-WS and CAR-Mobile. CAR is compatible with both greyscale images and images with RGB channels, offering adjustable settings brightness, contrast, and a detailed color map. During reconstruction, users have the flexibility to shift, rotate, or scale 3D objects for optimal observation. Furthermore, CAR allows users to explore adjacent image blocks or blocks at different resolutions while examining ultra-large-scale whole-brain images.

CAR-WS offers versatile rendering options for multidimensional image data, including maximum intensity projection, alpha-blending projection, cross-sectional views, and arbitrary cutting planes. It supports color mapping for different fluorescent channels, enhancing visualization. Users can interactively rotate, zoom, and pan using the mouse and delineate the skeletal structure of the neuron within a three-dimensional setting. The 3D visualization and annotation capabilities are powered by the virtual finger algorithm (Peng, et al., 2014), generating intricate 3D prior information that guides image analysis and proof edits the results of image computation.

CAR-VR is built upon OpenVR and thus should work for any compatible VR devices and operating systems. As shown in our implementation, we use tethered VR devices such as HTC Vive and HTC Vive Pro. CAR-VR supports a diverse range of functionalities for visualization, such as immersive 3D navigation, image adjustment, volume cutting, object showing/hiding, and more. In terms of annotation, it supports segment annotation, deletion, editing, subdivision, splitting, marker annotation, deletion, undo/redo, and other related features.

CAR-Mobile is a novel tool that offers convenience for users requiring mobility and immediate interactivity of the data. It supports real time interactive visualization with large scale bioimage data, such as mesoscopic whole-brain data. CAR-Mobile can navigate image block easily by one click, permit rotate, zoom in and out image just by finger scaling. It supports annotation functions like pinpointing, drawing curve, splitting curve, deleting curve/marker, deleting multi-curve/marker, clearing all annotation, changing curve/marker type and so on. In order provide smooth user experience, CAR-Mobile includes an optimized virtual finger algorithm with lower computation costs. The optimized algorithm initially calculates a rough curve, displayed on the screen, while a more complex algorithm runs on a child thread. Once completed, the accurate result replaces the rough one, streamlining the entire process for improved fluency.

2 CAR Server

CAR server is used to manage data and coordinate collaborations across multiple clients. The server is developed with Golang and C++. The server utilizes Docker container technology for deployment, comprising multiple containers for various server programs. These containers are interconnected through Docker Network for communication between different containers. The server consists of the following modules: service entry module, core server module, data management module and AI module.

The service entry module, composed of a Nginx container, is tasked with managing all HTTP requests. It processes these requests by parsing the URL. If it is a request for a file resource, it returns the static resource file. Otherwise, the request is forwarded to the core server module for further processing.

The core server module consists of a core server container, a MySQL database container, and a Redis data caching container. The core server has two components: an HTTP Server implemented in Golang and a collaborative server (implemented in C++). The HTTP Server handles all HTTP requests forwarded by the service entry module, performs the vast majority of read and write operations on MySQL/Redis containers, and invoke the collaborative server. The collaborative server manages multi-client collaboration, including operation processing, and data synchronization. It collaborates with the data management module in CAR to resolve conflicts and ensure data consistency after multi-client collaboration. The collaboration process begins with the creation of collaboration rooms, where each room corresponds to a specific reconstruction task. We employ mutex locks between annotation tasks to guarantee secure access to shared resources by various threads, mitigating read-write conflicts and upholding data reliability. In one collaboration room, it establishes a TCP Socket connection with all connected clients. Its main role is to receive and process annotation operation instructions from clients and broadcast them to other clients within the same collaboration task. This ensures that everyone has access to the same information in real-time.

The MySQL database container stores image metadata (image ID, coordinates, resolution information, etc.). The Redis data caching container is employed to cache data requests for information that needs to be frequently read, including user authentication details, image metadata, and so on.

Data Management

The data management module in CAR manages all collaborative operation data, encompassing upload and download processes, and incorporates version control based on timestamps. Users can utilize the front-end interface directly from the CAR-WS client for importing and exporting reconstruction data in

batch and tracing back to historical reconstruction data at any desired moment. It also offers functionalities for user data management and authentication.

1. User Authentication and Authorization

User authentication relies on a token-based method, generating a unique token upon successful login for subsequent session identification. Token validity is ensured through client-server heartbeat exchanges. Token expiration is marked if no heartbeat is received within a set time or upon exceeding the lifespan, requiring reauthentication. This approach offers statelessness, scalability, and high security. For data access control, a permission management system based on groups is implemented, along with a project-based system for customizing access permissions, ensuring isolation and security for team-generated data. Denied requests result when users lack specific operation permissions.

2. Client-Server Communication

We adopt the Remote Procedure Call (RPC) approach for communication between different CAR clients (VR/Mobile/Workstation) and CAR server.

In detail, we use gRPC, an open-source RPC framework based on Protocol Buffers (protobuf) and HTTP/2. gRPC facilitates communication through proto files, defining the messages to be transmitted and the methods of remote procedure calls. It generates code for various programming languages using the protoc tool, enabling developers to invoke the corresponding interfaces without concerning about underlying details. In addition, as gRPC lacks native browser support, we introduce a reverse proxy service for HTTP requests in CAR server, translating them into corresponding gRPC requests. This enables CAR to flexibly support both gRPC and HTTP communication, increasing its compatibility.

3. Imaging data storage

CAR is mostly used for multidimensional light microscopic images and is not limited to a specific image type. Also, there is virtually no limit on the resolution of the image data, as long as there is enough storage. Image data in CAR can be stored locally on each user’s system, as well as on the CAR server. Alternatively, a shared copy can be hosted on a web data storage accessible by both the CAR clients and the CAR server.

4. Morphological data storage

MongoDB is used to store reconstructed morphological data (SWC file). The SWC file format is enriched with more information about each node when storing it in the database, such as creation date and creator information. This allows for more detailed and efficient management of morphology data, combined with version control and conditional query features. With version control, the user can revert the reconstruction data to a historical moment. Using conditional queries, the user can select and export specific reconstruction data based on specified dates and creators.

5. Version control system

As morphological data is crucial throughout the production process, we introduce a version control system for neuron morphology data to save and trace neuron data at different time points. This system performs periodic full backups of morphology data, which can also be manually initiated. We use incremental operation records between every two full backups. Every operation on morphology data, such as addition, modification, or deletion, is recorded. Clients can trace back to any moment to obtain historical morphological data. The combination of full and incremental backups not only records all morphology data from creation but also mitigates the space occupation problem caused by full backups.

3 Programming environments

CAR-Server is programmed with Golang and C++. In particular, the C++ code is dedicated to the coordination of collaborative annotation among users, while the Golang codes are responsible for handling connection requests and database access.

CAR-WS and CAR-VR are developed using C/C++, with the Qt library employed for the development of the user interface and OpenVR used as the software development kit for connection to VR hardware. CAR-Mobile is developed using Java and the Android software development kit. For CAR-Game, it is developed using C# on the Unity platform.

4 Hardware and environment configurations

CAR server is deployed on a server with the below configurations:
- CPU: Intel(R) Xeon(R) Platinum 8255C CPU @ 2.50GHz
- GPU: 1 * NVIDIA Tesla V100
- Memory: 80GB
- Storage: 500GB SSD and 1TB HDD
- Network: 100Mbps
- Operating System: Ubuntu 20.04 LTS

The mainly adopted environment configurations for CAR server include:
- Number of maximal concurrent connections: 200

- Number of maximal concurrent jobs: 20
- Interval for AI module inference (seconds): 180

CAR-WS, CAR-VR, CAR-Game are recommended to run on a workstation with the below configurations:
- CPU: Intel(R) Core(TM) i7-8700 CPU @ 3.20GHz
- GPU: NVIDIA GeForce GTX 1070
- Memory: 64GB
- Storage: 500GB
- VR headset (for CAR-VR): HTC Vive or Vive Pro

CAR-Mobile is recommended to run on a mobile phone with the below configurations:
- CPU: Qualcomm Snapdragon 855
- Memory: 8GB
- Storage: 128GB

5 Supplementary Tables

Supplementary Table 1 | A comparison between CAR and other software tools for neuron reconstruction. In detail, we evaluated their capabilities on collaborative reconstruction and support for different kinds of devices. "—" indicates that the function is not supported by the specific tool.

		Amira	Vaa3D	Neurolucida	SNT	Janelia Workstation	CATMAID	Neuroglancer	CAR
Reconstruction		LM	LM	LM	LM	LM	EM	EM	LM
	**Annotation approach**	3D	3D	3D	2D slice-by-slice	3D	2D slice-by-slice	2D slice-by-slice	Immersive 3D
	**Multi-resolution visualization**	Yes	Yes	—	—	Yes	—	—	Yes
	**3D collaborative editing**	—	—	—	—	—	—	—	Yes
	**Cross-device collaboration**	—	—	—	—	—	—	—	Yes
	**AI collaborator**	—	—	—	—	—	—	—	Yes
Device support	**Desktop workstations**	Yes	Yes		Yes	Yes	Yes	Yes	Yes
	**VR headsets**	—	Supported via TeraVR.	—	Supported via third party. Visualization only.	—	—	—	Yes
	**Mobile phones**	—	—	—	—	—	—	—	Yes
**Reported large-scale applications to both mouse and human neurons**		—	Both Yes	—	—	Mouse	—	—	Both Yes
**Reference / link**		Stalling, et al., 2005	Peng, et al., 2014a	Glaser, et al.,1999	Arshadi, et al.,2021	Winnubst, et al., 2019	Schneider-Mizell, et al., 2016	github.com/google/neuroglancer	This study

Supplementary Table 2 | Features and a list of typical supported applications for each CAR components.

Component	Features	Applications
CAR-WS	CAR-WS has functionality and collaborative features designed for whole neuron reconstruction. It allows annotating and visualizing neuronal structures in their entirety.	3D brain region tagging,<br>complete neurons reconstruction,<br>automatic neuron reconstruction,<br>soma identification,<br>bouton verification,<br>neuron morphometry measurements,<br>management of the server-side annotation data.
CAR-VR	CAR-VR offers synchronized real-time stereo rendering, providing an environment for annotation and correcting errors in multi-dimensional observations.	Complete neurons reconstruction, especially at challenging regions,<br>reconstruction proofreading,<br>soma identification,<br>bouton verification.
CAR-Mobile	CAR-Mobile provides access to data using mobile-phones, enables quick interaction and annotation of the multi-dimensional data, and facilitates large-scale, low-cost collaboration.	Image quality evaluation,<br>simple neuronal structure reconstruction,<br>automatic neuron reconstruction,<br>soma identification,<br>bouton verification.
CAR-Game	CAR-Game currently provides a First-Person Shooter (FPS) game for crowdsourcing.	Neuronal topology proofreading.

Supplementary Table 3 | Major distinctions between mouse and human brain images

	**Mouse brain images**	**Human brain images**
Imaging approach	fMOST	Two-photon
Labeling approach	Sparsely labeling by virus injection	Fluorescent labeling through cell injection
Image size	Whole-brain scales (~ 40,000×30,000×10,000 voxels)	Local regions (~ 512×512×128 voxels)
Resolution	0.2-0.35 μm in the xy plane, 1 μm in the z direction	0.15~0.92 μm in the xy plane, 1 μm in the z direction
Soma radius	10~20 μm	15~30 μm
Morphology	Complete morphology, multiple neurons	Dendrites, single neuron
Signal quality	Having weak and broken signals	Having weak signals
Noise	Anisotropic noise	Consistently high noise levels

Supplementary Table 4 Measures of BPV and TPV classifiers

	BPV	TPV
**TP**	The number of branching points that exist in the GT and are classified as branching points	The number of terminal points that exist in the GT and are classified as terminal points
**TN**	The number of branching points that are missing in the GT and are hinted as non-branching points	The number of terminal points that are missing in the GT and are hinted as breakpoints
**FP**	The number of branching points that are missing in the GT but are classified as branching points	The number of terminal points that are missing in the GT but are classified as terminal points
**FN**	The number of branching points that exist in the GT but are hinted as non-branching points	The number of terminal points that exist in the GT but are hinted as breakpoints

6 Supplementary Figures

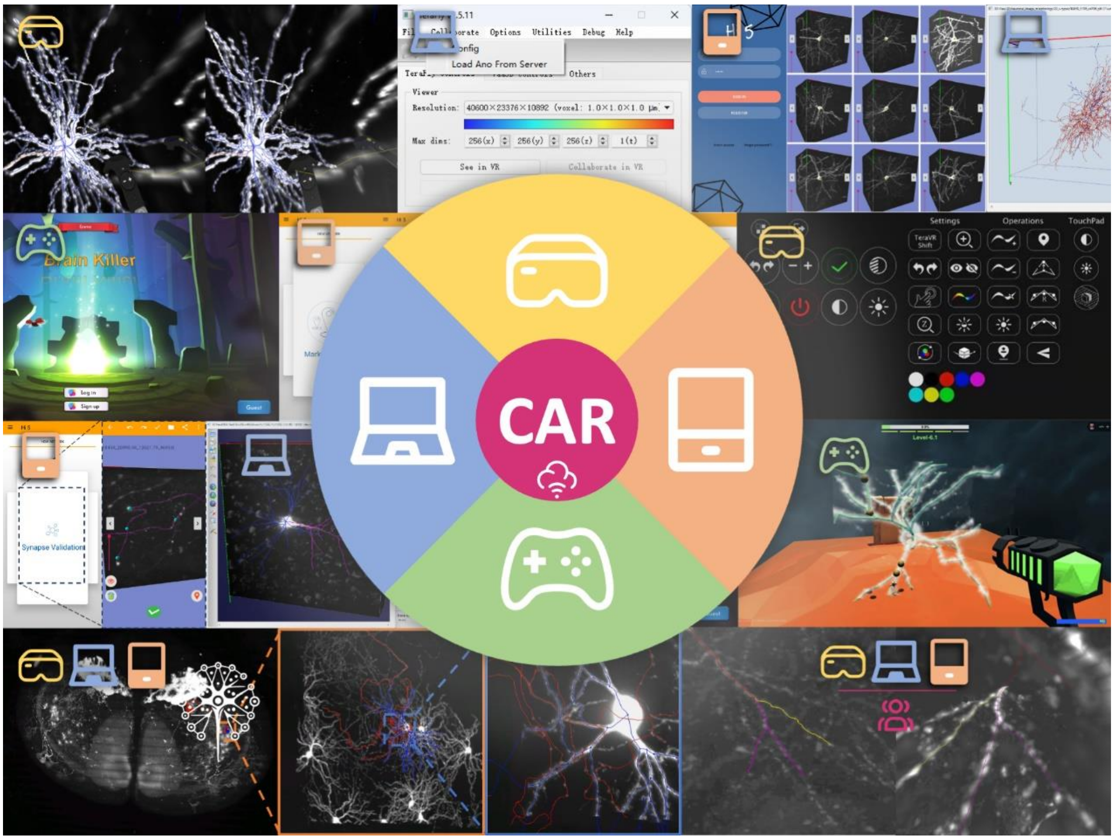

Supplementary Fig. 1 | Overview of the CAR client software user interface. The CAR platform offers an array of diverse client software, each equipped with the ability to seamlessly interact with other clients, whether of the same type or different, through the CAR server. With the workstation (depicted by a blue icon), the virtual reality tool (yellow), or even the mobile app (orange), users can jointly explore neuronal data across multiple levels of resolution and execute various neuromorphometric tasks. Additionally, users may further access and curate neuronal data through the CAR game console (green).

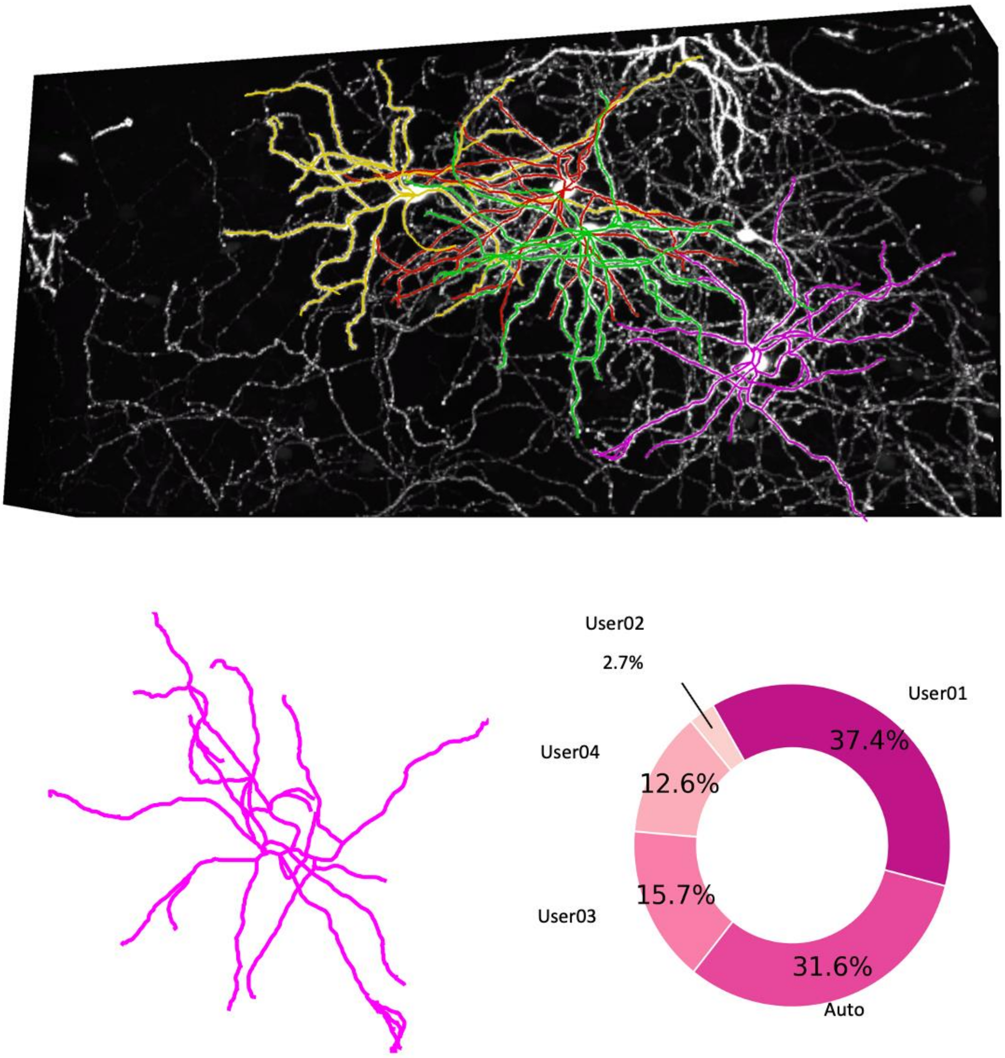

Supplementary Fig. 2 | Collaboration efforts on multiple reconstruction tasks. Top: In the showcased brain image, a team of four users collaborated on the simultaneous reconstruction of four neurons, aided by the automatic tracing module integrated within CAR. Bottom: The morphology of one reconstructed neuron, together with a chart demonstrating the contribution ratio (in terms of reconstructed length) of the four users and the automation module.

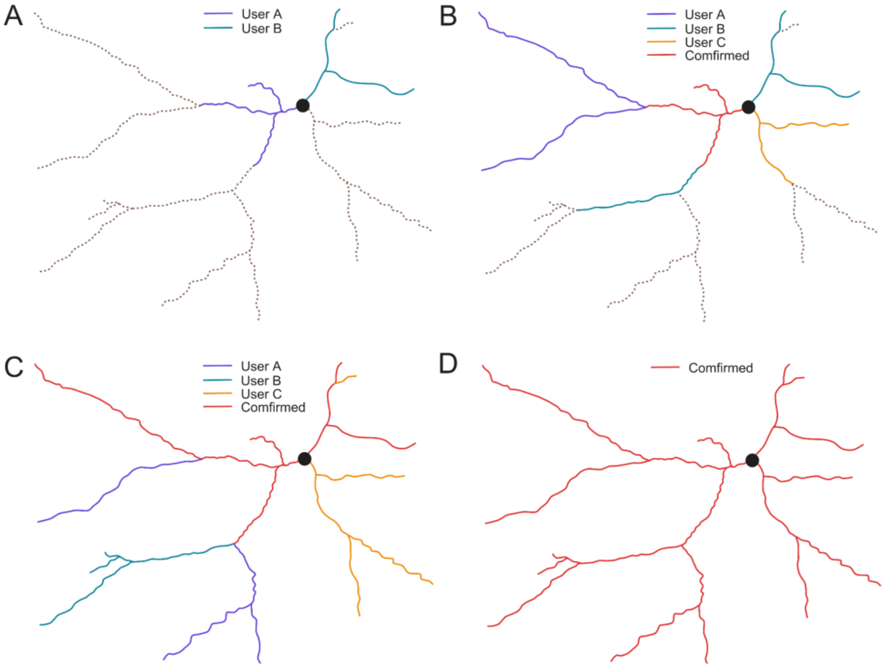

Supplementary Fig. 3 | Collaborative neuron reconstruction protocol. A, **B**, **C**, **D**, four subsequent stages during the reconstruction of a neuron. Purple, green, and orange lines indicate neurites reconstructed by 3 different users, respectively. Red lines represent confirmed neurites. Dashed lines represent neurites that are yet reconstructed at the moment. A reconstruction is considered “finished” when all structures have been annotated and confirmed.

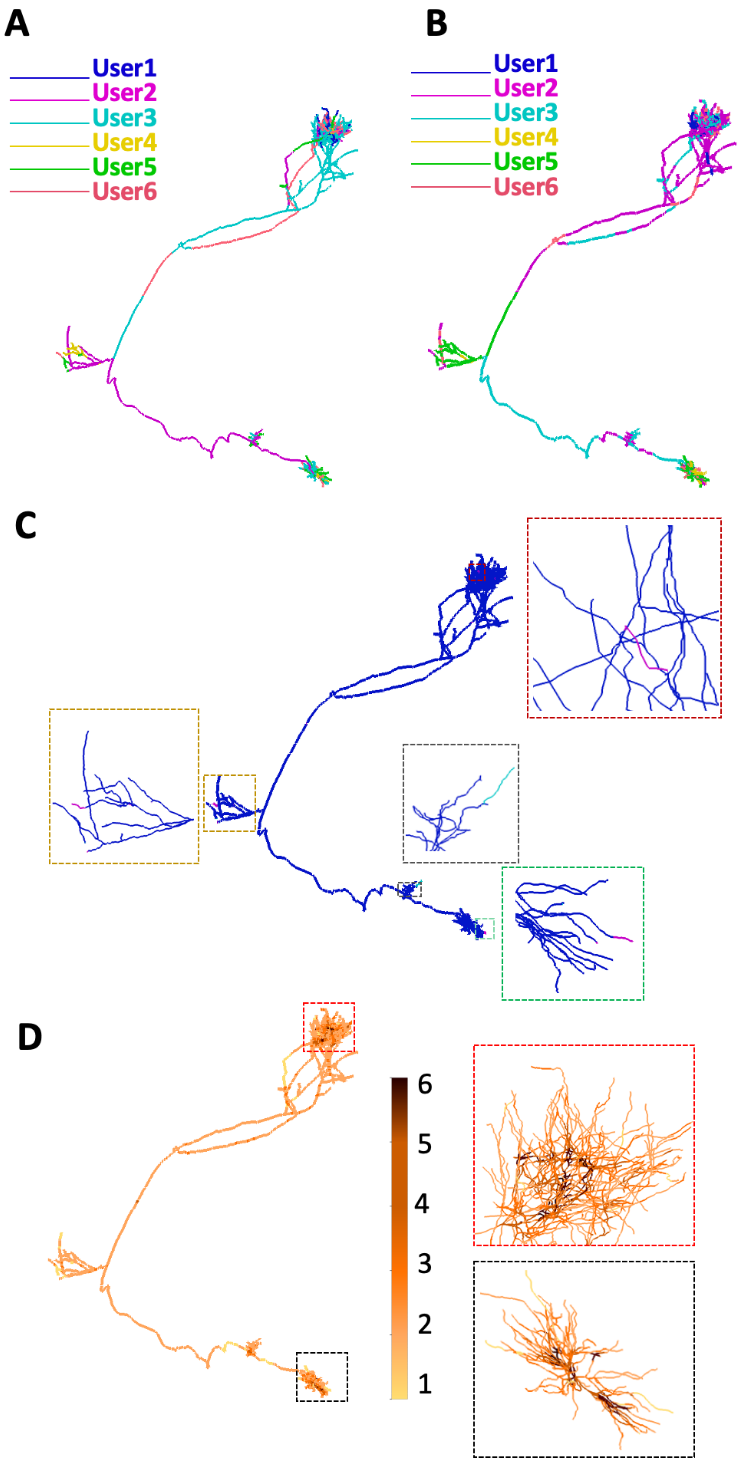

Supplementary Fig.4 | A CAR-reconstructed VPL neuron. A, Visualization of the neuron, with each user's annotations represented in distinct colors. **B**, Visualization of the neuron, showcasing each user's proofreading efforts in distinct colors. **C**, Expert modifications to the citizen scientists' consensus result. Neurites deleted by the expert are marked in magenta, while neurites added by the expert are highlighted in cyan. **D**, A heatmap illustrating user participation across various regions of the neuron. The number of participating users is color-coded according to the color map.

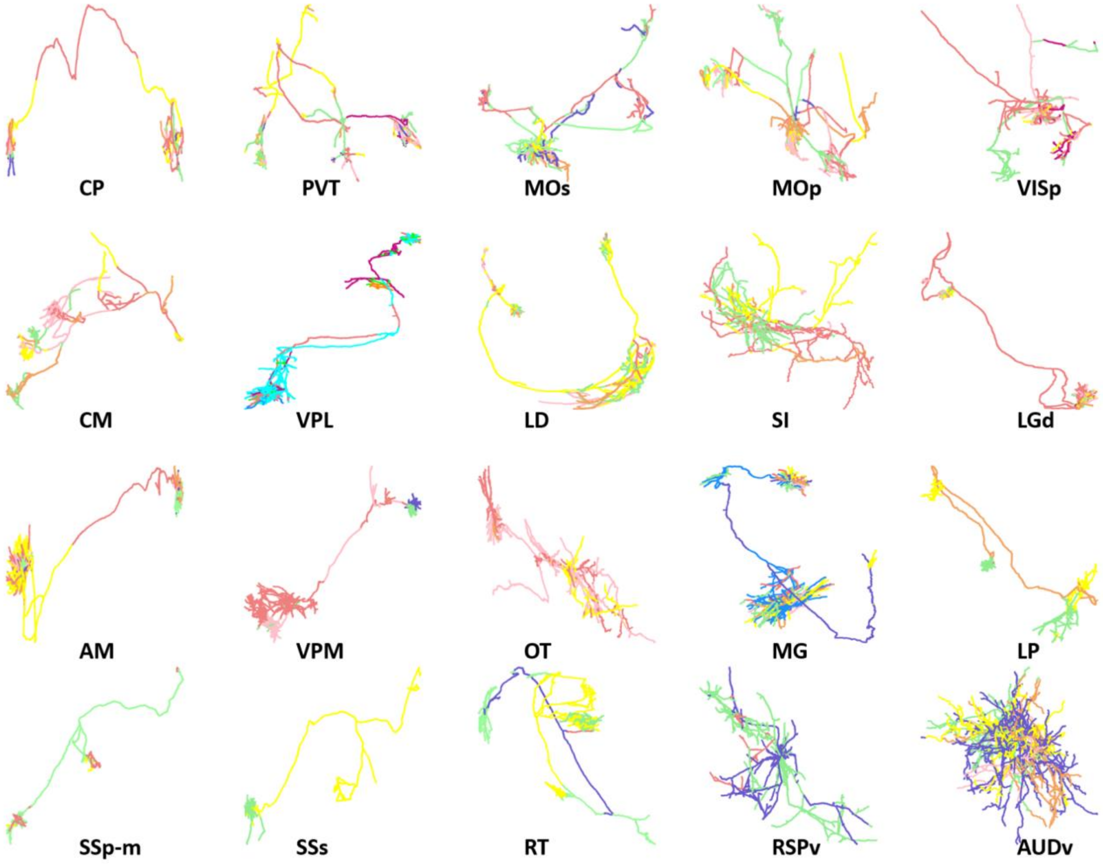

Supplementary Fig. 5 | Collaborative reconstruction of 20 mouse neurons using CAR. In this collaborative effort, 12 individual users contributed to the reconstruction of 20 mouse neurons, while the number of contributors for each neuron varies. For an evaluation of their collaborative synergy, each user was assigned a unique color, and the neurons were color-coded accordingly to emphasize different users' contributions. Additionally, the validation process may involve more users, who cross-check and verify neurite annotations made by others.

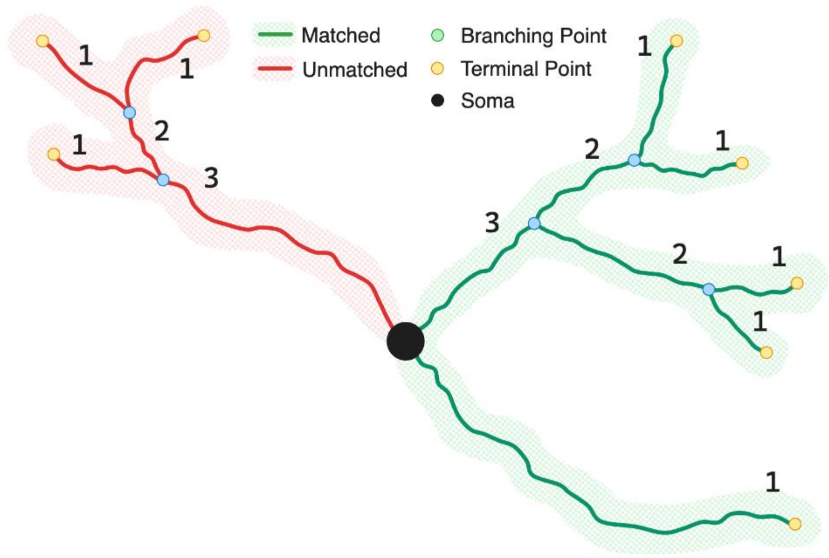

Supplementary Fig. 6 | Illustration of the calculation of normalized topological height (NTH). Segments adjacent to terminal points are assigned a topological height of 1. For any other segment, its topological height is defined as one plus the greater topological height of its two sub-segments. Eventually, the topological height values are normalized by dividing them by the maximum topological height.

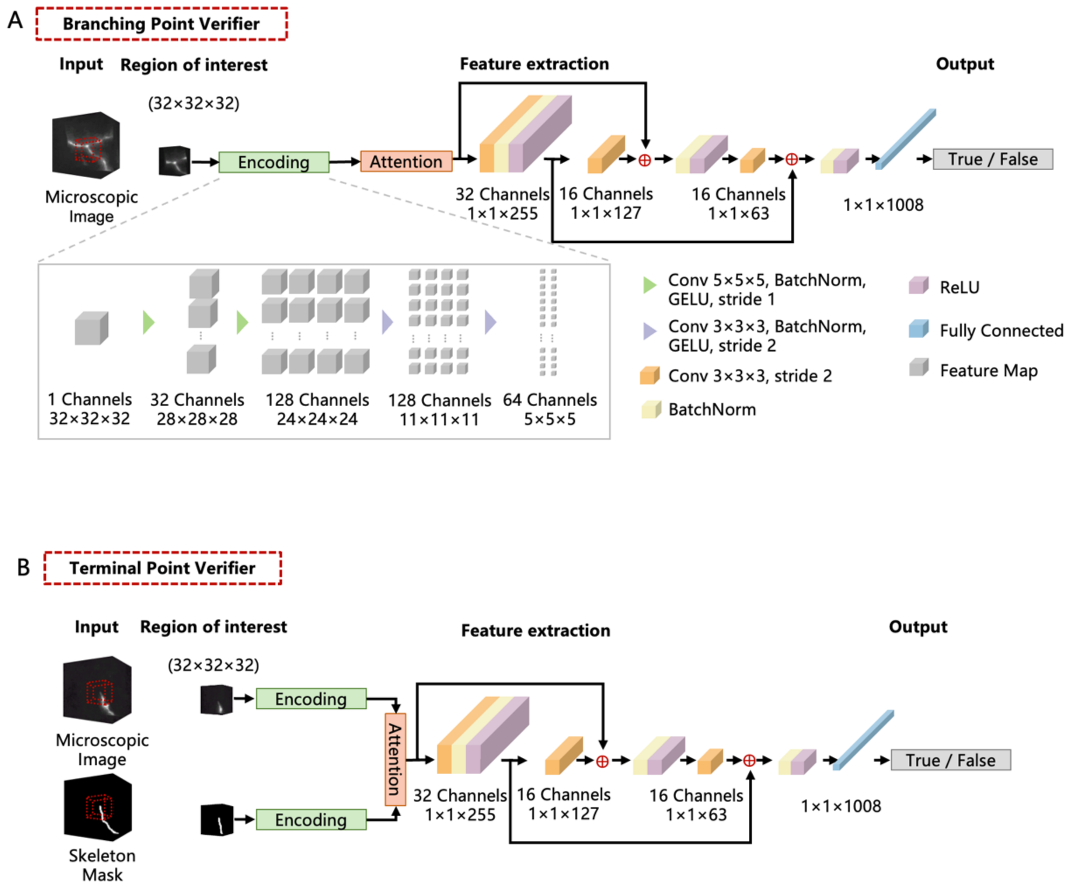

Supplementary Fig. 7 | Network architectures of Branching Point Verifier and Terminal Point Verifier. A, The Branching Point Verifier is designed as a Residual Single Head Network (RSHN), comprising a encoding module, an attention module, and two residual blocks. Details regarding dimension and channel encoding are illustrated in the inset. **B**, The **Terminal Point Verifier** uses Residual Single Head Network (RDHN), which is similar as RSHN with the distinction of accepting two inputs: an image patch extracted around potential terminal points and a corresponding mask image derived from the reconstruction.

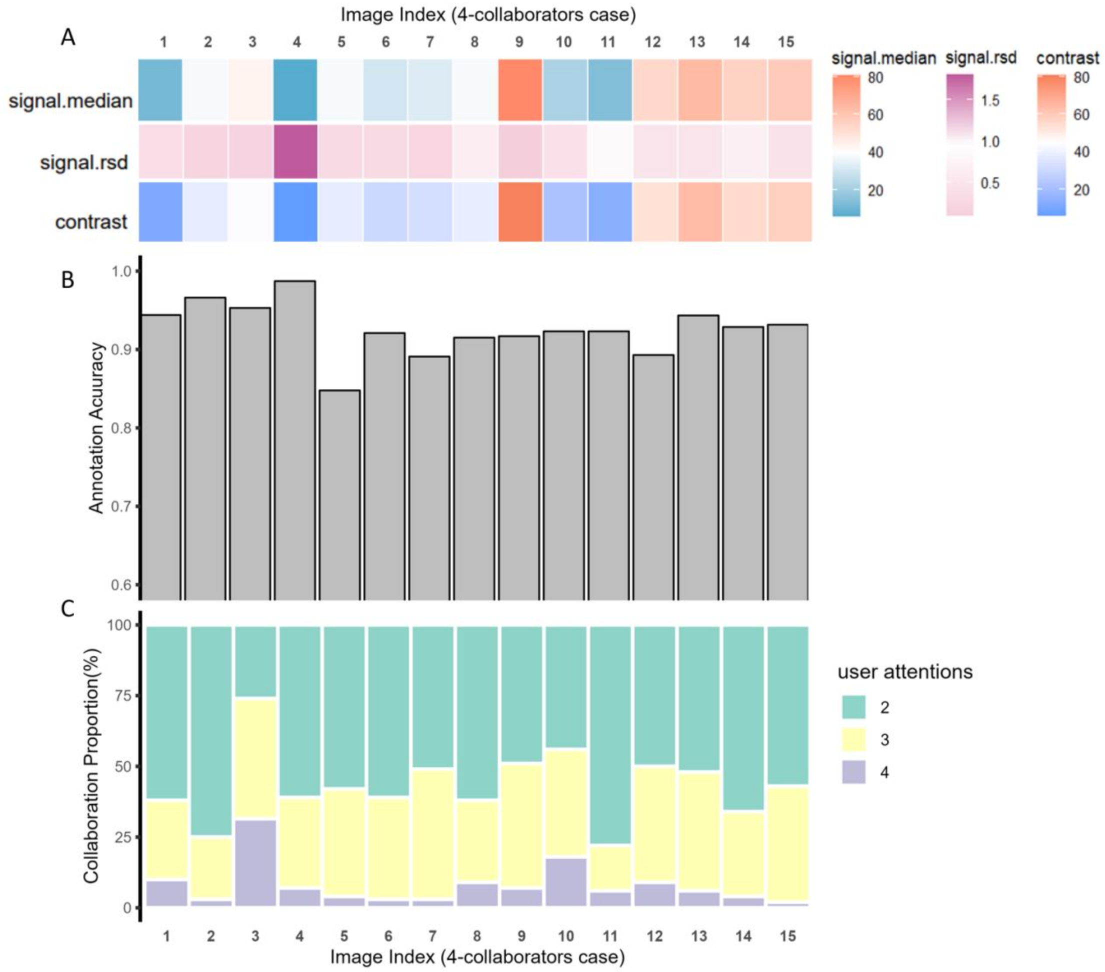

Supplementary Fig. 8 | Assessment of image quality, reconstruction accuracy, and user attentions for neurons reconstructed by 4-collaborator groups. A, Characteristics of the 15 individual image data used by 4-collaborator groups. First row (signal.median): the median intensity of the signal (foreground). Second row (signal.rsd): the relative standard deviation (rsd) of the signal (foreground). Third row: the contrast of the image (**Methods**). **B**, The reconstruction accuracy of the 15 neurons. **C**, The degree of collaboration is illustrated using color coding: green represents the proportion of reconstructions conducted by 2 collaborators, yellow signifies 3 collaborators, and purple denotes 4 collaborators.

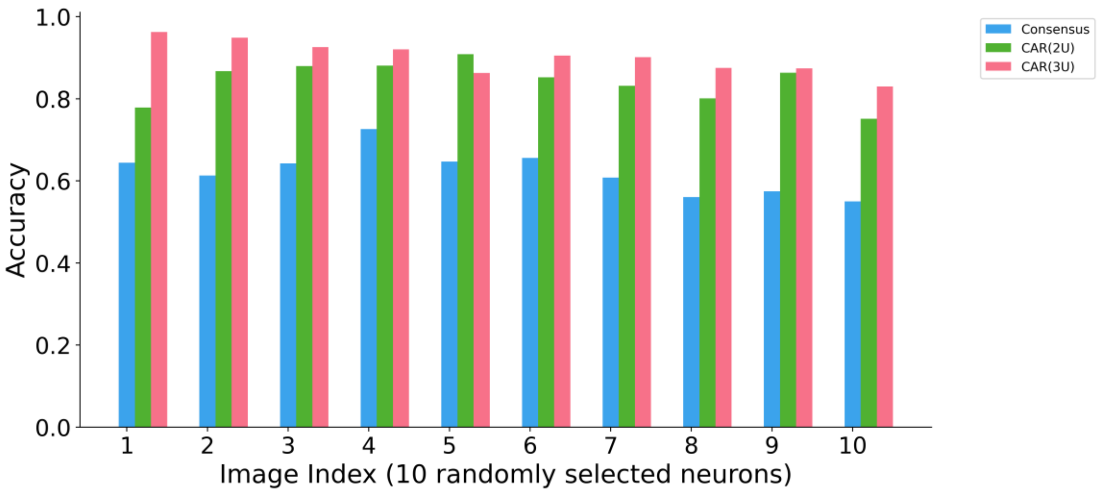

Supplementary Fig. 9 | Accuracy of the reconstructions for 10 human neurons. For each image, a consensus result (blue) was produced based on combining the Vaa3D and SNT reconstructions already analyzed in **Fig. 4D** and two additional, independently generated reconstructions using Vaa3D. The reconstruction accuracies using CAR with 3 users (pink), CAR with 2 users (green), and the consensus approach (blue) are then illustrated.

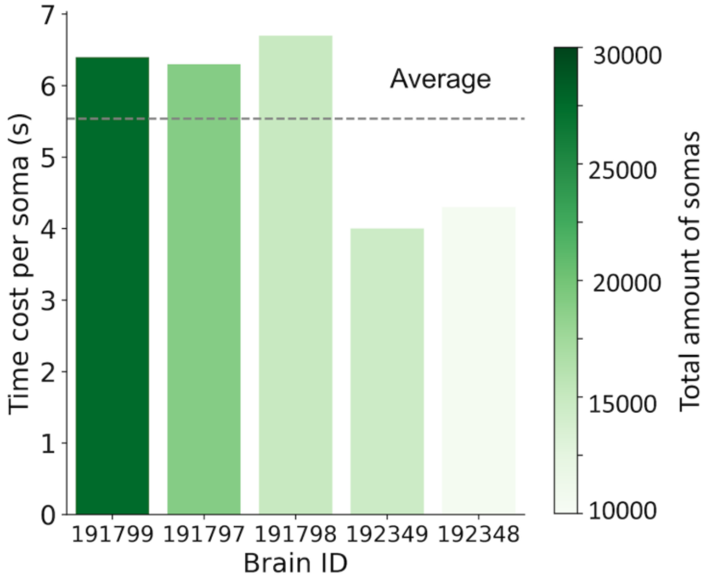

Supplementary Fig. 10 | Time cost of soma annotation for five whole-brain image datasets. The height of each bar illustrates the average time cost for the annotation of the somas in the specific brain. The dashed line in the figure indicates the average annotation time, which is 5.5 seconds, for each soma. The colors of the bars indicate the total numbers of annotated somas in these brains.
