# Supplementary material for: Collaborative augmented reconstruction of 3D neuron morphology in mouse and human brains
Source: Nat Methods. 2024 Sep 4;21(10):1936–46. doi: 10.1038/s41592-024-02401-8 (PMC11468770; doi:10.1038/s41592-024-02401-8)
Supplement: Supplementary file 2 — Reporting Summary [file 41592_2024_2401_MOESM2_ESM.pdf]

nature portfolio

Corresponding author(s): Hanchuan Peng

Last updated by author(s): May 11, 2024

Reporting Summary

Nature Portfolio wishes to improve the reproducibility of the work that we publish. This form provides structure for consistency and transparency in reporting. For further information on Nature Portfolio policies, see our Editorial Policies and the Editorial Policy Checklist.

Statistics

For all statistical analyses, confirm that the following items are present in the figure legend, table legend, main text, or Methods section.

n/a	Confirmed	
☐	☒	The exact sample size (*n*) for each experimental group/condition, given as a discrete number and unit of measurement
☐	☒	A statement on whether measurements were taken from distinct samples or whether the same sample was measured repeatedly
☒	☐	The statistical test(s) used AND whether they are one- or two-sided<br>*Only common tests should be described solely by name; describe more complex techniques in the Methods section.*
☒	☐	A description of all covariates tested
☐	☒	A description of any assumptions or corrections, such as tests of normality and adjustment for multiple comparisons
☐	☒	A full description of the statistical parameters including central tendency (e.g. means) or other basic estimates (e.g. regression coefficient) AND variation (e.g. standard deviation) or associated estimates of uncertainty (e.g. confidence intervals)
☒	☐	For null hypothesis testing, the test statistic (e.g. *F*, *t*, *r*) with confidence intervals, effect sizes, degrees of freedom and *P* value noted<br>*Give P values as exact values whenever suitable.*
☒	☐	For Bayesian analysis, information on the choice of priors and Markov chain Monte Carlo settings
☒	☐	For hierarchical and complex designs, identification of the appropriate level for tests and full reporting of outcomes
☒	☐	Estimates of effect sizes (e.g. Cohen's *d*, Pearson's *r*), indicating how they were calculated

Our web collection on statistics for biologists contains articles on many of the points above.

Software and code

Policy information about availability of computer code

Data collection	All the data reported in this manuscript, including neuron reconstructions, soma locations, and synaptic sites, were generated using the Collaborative Augmented Reconstruction (CAR) platform (version: 1.3.1).
Data analysis	As a data postprocessing step after reconstruction, registration was performed using the updated version of mBrainAligner, which can be found at https://github.com/Vaa3D/vaa3d_tools/tree/master/hackathon/mBrainAligner. Other necessary postprocessings of the reconstructions were performed using Vaa3D (version 4.001, https://github.com/Vaa3D),Terafly(version 2.5.11) and its plug-ins. The mouse neuron projection patterns were quantified using the python package neuro_morpho_toolbox (python version 3.8), which is available at https://github.com/pengxie-bioinfo/neuro_morpho_toolbox. The custom code developed in the study is avaliable at https://github.com/neurogeom/CAR.

For manuscripts utilizing custom algorithms or software that are central to the research but not yet described in published literature, software must be made available to editors and reviewers. We strongly encourage code deposition in a community repository (e.g. GitHub). See the Nature Portfolio guidelines for submitting code & software for further information.

Data

Policy information about availability of data

All manuscripts must include a data availability statement. This statement should provide the following information, where applicable:

- Accession codes, unique identifiers, or web links for publicly available datasets
- A description of any restrictions on data availability
- For clinical datasets or third party data, please ensure that the statement adheres to our policy

All the data reported in this manuscript, including mouse and human neuron reconstructions, soma locations, and synaptic sites, are deposited to https://car.cvcd.xyz.

Human research participants

Policy information about studies involving human research participants and Sex and Gender in Research.

Reporting on sex and gender	N/A
Population characteristics	N/A
Recruitment	N/A
Ethics oversight	N/A

Note that full information on the approval of the study protocol must also be provided in the manuscript.

Field-specific reporting

Please select the one below that is the best fit for your research. If you are not sure, read the appropriate sections before making your selection.

☒ Life sciences ☐ Behavioural & social sciences ☐ Ecological, evolutionary & environmental sciences

For a reference copy of the document with all sections, see nature.com/documents/nr-reporting-summary-flat.pdf

Life sciences study design

All studies must disclose on these points even when the disclosure is negative.

Sample size	In Figure 2, we selected mouse projection neurons from 20 distinct brain regions, one from each, ensuring a diverse representation of cell types, projection patterns, and morphologies. These neurons, derived from various transgenic lines, contribute to a broad range of distinct morphological features and are comprehensively used to evaluate neuronal reconstruction. In Figure 4, we utilized 80 human neurons sourced from 10 anatomical regions. This sample size was chosen based on similar principles, aiming to provide a comprehensive overview and ensure diverse coverage. For Figure 5, the sample size included 156,000 annotated somas, selected from 58 whole brains and 609 brain regions, facilitating a robust validation of the method's reliability on a large scale. Overall, the choice of these sample sizes was strategically made to ensure the broad applicability.
Data exclusions	No data were excluded for the analysis.
Replication	All the experiments in this work involved multiple pieces of testing data, which were combined for analysis. Results for such analysis always showed a clear and consistent pattern. This indicates that the work has good reproducibility.
Randomization	For all the involved experiments, people collaborated freely to generate morphological data. There was no special consideration for assigning annotators to a neuron, and the allocation of annotators can be in fact considered as random. For the human neuron-related experiments, data were divided, on a completely random basis, into several groups in order to compare the performance among different tools/settings.
Blinding	The investigators were blinded to the data annotators' independent work on data collection. The investigators were also blinded to group allocation during the data analysis phase of the study.

Reporting for specific materials, systems and methods

We require information from authors about some types of materials, experimental systems and methods used in many studies. Here, indicate whether each material, system or method listed is relevant to your study. If you are not sure if a list item applies to your research, read the appropriate section before selecting a response.

Materials & experimental systems

n/a	Involved in the study
☒	☐ Antibodies
☒	☐ Eukaryotic cell lines
☒	☐ Palaeontology and archaeology
☒	☐ Animals and other organisms
☒	☐ Clinical data
☒	☐ Dual use research of concern

Methods

n/a	Involved in the study
☒	☐ ChIP-seq
☒	☐ Flow cytometry
☒	☐ MRI-based neuroimaging
